# Somatic Hypermutation and Framework Mutations of Variable Region Contribute to Anti-Zika Virus-Specific Monoclonal Antibody Binding and Function

**DOI:** 10.1128/jvi.00071-22

**Published:** 2022-05-16

**Authors:** Isamu Tsuji, Fue Vang, David Dominguez, Lovkesh Karwal, Ankita Sanjali, Jill A. Livengood, Edgar Davidson, Mallorie E. Fouch, Benjamin J. Doranz, Subash C. Das, Hansi J. Dean

**Affiliations:** a Vaccine Business Unit, Takeda Pharmaceutical Ltd., Cambridge, Massachusetts, USA; b Integral Molecular, Philadelphia, Pennsylvania, USA; University of North Carolina at Chapel Hill

**Keywords:** Zika, antibody function, affinity maturation, somatic hypermutation, framework region mutation, Zika virus structure, correlation analysis, neutralizing antibodies

## Abstract

Zika virus (ZIKV) is a global public health concern due to its ability to cause congenital Zika syndrome and lack of approved vaccine, therapeutic, or other control measures. We discovered eight novel rabbit monoclonal antibodies (MAbs) that bind to distinct ZIKV envelope protein epitopes. The majority of the MAbs were ZIKV specific and targeted the lateral ridge of the envelope (E) protein domain III, while the MAb with the highest neutralizing activity recognized a putative quaternary epitope spanning E protein domains I and III. One of the non-neutralizing MAbs specifically recognized ZIKV precursor membrane protein (prM). Somatic hypermutation of immunoglobulin variable regions increases antibody affinity maturation and triggers antibody class switching. Negative correlations were observed between the somatic hypermutation rate of the immunoglobulin heavy-chain variable region and antibody binding parameters such as equilibrium dissociation constant, dissociation constant, and half-maximal effective concentration value of MAb binding to ZIKV virus-like particles. Complementarity-determining regions recognize the antigen epitopes and are scaffolded by canonical framework regions. Reversion of framework region amino acids to the rabbit germ line sequence decreased anti-ZIKV MAb binding activity of some MAbs. Thus, antibody affinity maturation, including somatic hypermutation and framework region mutations, contributed to the binding and function of these anti-ZIKV MAbs.

**IMPORTANCE** ZIKV is a global health concern against which no vaccine or therapeutics are available. We characterized eight novel rabbit monoclonal antibodies recognizing ZIKV envelope and prM proteins and studied the relationship between somatic hypermutation of complementarity-determining regions, framework regions, mutations, antibody specificity, binding, and neutralizing activity. The results contribute to understanding structural features and somatic mutation pathways by which potent Zika virus-neutralizing antibodies can evolve, including the role of antibody framework regions.

## INTRODUCTION

Zika virus (ZIKV) is a flavivirus that is transmitted to humans through mosquitoes (1) and can also be transmitted between humans through sexual contact (2) and vertically through pregnancy (3). ZIKV was initially identified in Africa in 1947 (4). Epidemics were reported in Micronesia in 2007 (5) and French Polynesia in 2013 to 2014, with the virus subsequently spreading to other countries in Oceania (6, 7). While most ZIKV infections cause mild disease, in 2015, ZIKV spread rapidly in the Americas and caused clusters of microcephaly and other congenital malformations in infants born to women infected during pregnancy (8). Infection has been associated with microcephaly and other developmental abnormalities in fetuses and newborn babies (9) and Guillain Barre syndrome, brain ischemia, myelitis, and meningoencephalitis in adults (8, 10). In February 2016, the World Health Organization (WHO) declared ZIKV a Public Health Emergency of International Concern (8, 11, 12). The number of ZIKV patients subsequently declined (13). However, ZIKV circulation has also been detected in numerous Asian and African countries (14), including India (15), Thailand (16), Malaysia (17), Myanmar (18), Angola (14), Kenya (19), Mali (20), and Ethiopia (21); thus, the virus still poses a public health threat (13). No vaccines or therapeutics are available to prevent or treat ZIKV infection or disease.

ZIKV is a positive-stranded RNA virus, closely related to other flaviviruses, including Dengue virus (DENV), Yellow fever virus, West Nile virus, and Japanese encephalitis virus (22). The viral genome is translated into a single polyprotein posttranslationally cleaved by cellular and viral proteases into three structural proteins, capsid, precursor membrane (prM), and envelope (E), and seven nonstructural proteins. ZIKV E protein is the primary immunological determinant for inducing neutralizing antibodies and consists of three domains: a central β-barrel domain (domain I [DI]), an extended finger-like dimerization domain (DII), and an immunoglobulin-like segment (DIII) (23). The distal end of DII contains the fusion loop (FL), a hydrophobic sequence that inserts into the host cell endosomal membrane during pH-dependent conformational changes that drive fusion of the viral and cellular membrane. In immature virions, ZIKV E protein forms a complex with the prM protein, which is cleaved in the *trans*-Golgi network, facilitating E protein rearrangement during virion maturation (24).

The human antibody repertoire is highly diverse due to the ability to randomly assemble variable (V), diversity (D), and joining (J) segments of immunoglobulin genes in B cells (25) during antibody affinity maturation. Antibody affinity maturation functions to increase antibody affinity and specificity, generating antibodies capable of effective antiviral activity (26). Affinity maturation is initiated by activation-induced cytidine deaminase, which promotes isotype switching by deaminating deoxycytidines within immunoglobulin genes, leading to somatic hypermutation (SHM) and class switch recombination (27). Immunoglobulin binding affinity and specificity are determined by the amino acids in the complementarity-determining regions (CDR), which generally form contacts with the antigen. Immunoglobulins contain six CDRs, three on the heavy-chain and three on the light-chain. During antibody affinity maturation, the CDRs undergo a high degree of somatic mutation. Among CDRs, the heavy-chain CDR3 (CDRH3), selected from the D allele, contains the highest degree of diversity in sequence and length (28, 29). The framework region (FWR) sequences, located between CDRs, form β barrel frameworks to stabilize the structure of the CDRs (27, 30, 31). While the FWR sequences are generally less tolerant of mutations, recently the accumulation of FWR mutations in anti-human immunodeficiency virus (HIV) antibodies was found to increase the breadth and potency of neutralizing antibodies, suggesting that the FWR can also contribute to antibody function (32–34).

Rabbit and human antibodies have similar features not shared by mouse antibodies in terms of B-cell ontogeny and diversity of antibody repertoire (35, 36). From these diverse repertoires, rabbit MAbs possess features such as high specificity (37), high affinity (38), and CDR3 regions that are similar in length to human CDR3s (39).

Here, we describe ZIKV-specific MAbs isolated after vaccination of rabbits with a combination of a purified inactivated Zika vaccine (PIZV) candidate and ZIKV virus-like particles (ZIKV-VLPs). The results demonstrate that both SHM and FWR mutations of anti-ZIKV MAbs contribute to antibody affinity, specificity, and functionality.

## RESULTS

### Binding and neutralization activity of anti-ZIKV MAbs.

Fourteen anti-ZIKV MAb clones were isolated from rabbits vaccinated with Takeda’s candidate PIZV and boosted with ZIKV-VLP. Based on preliminary screening and characterization, we selected eight MAbs with diverse characteristics (102-1, 242-3, 270-12, 289-3, 306-2, 78-2, 278-11, and 11-3) for further characterization.

**FIG 1:**
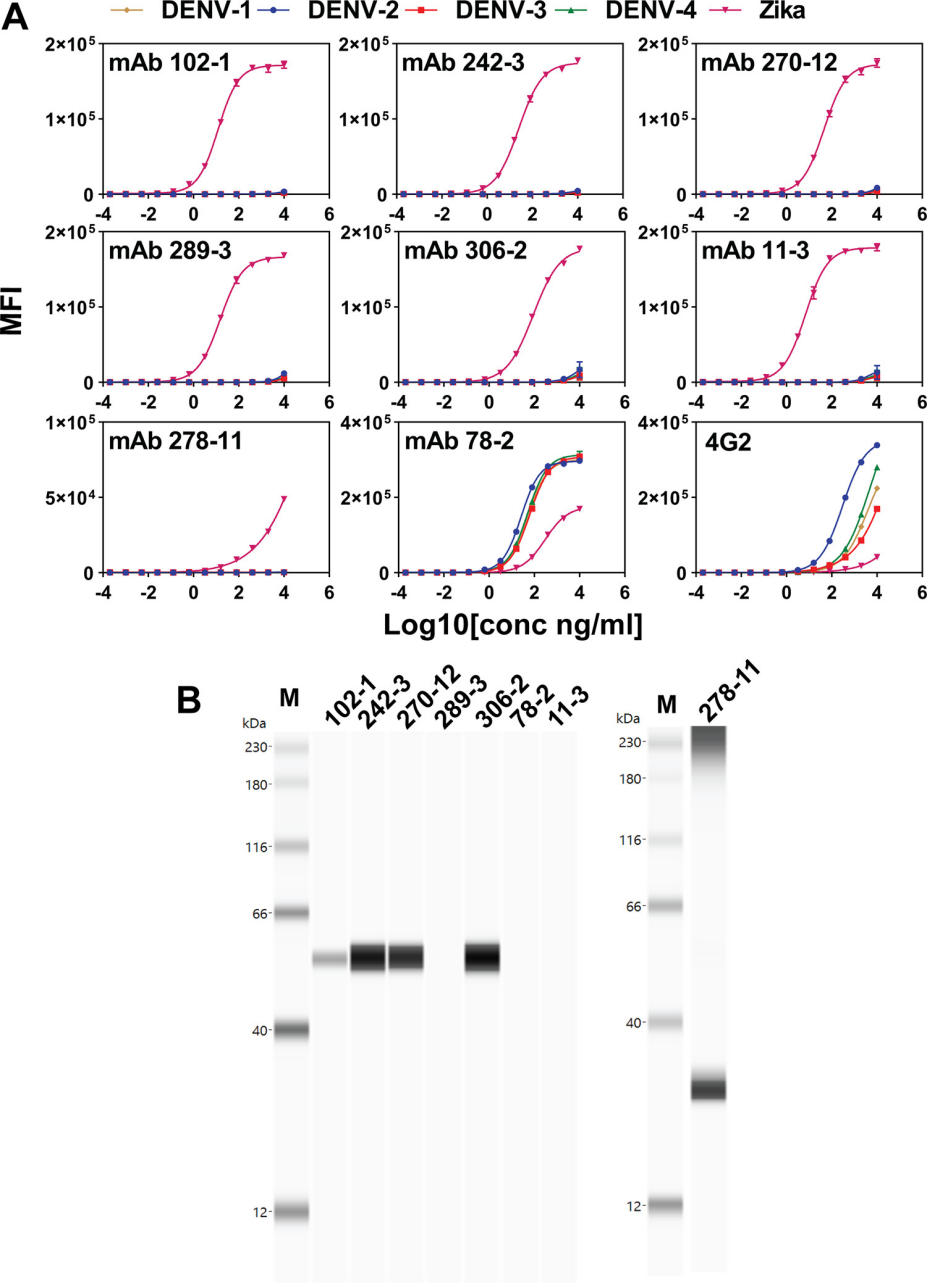
Reactivity of anti-ZIKV MAbs. (A) Reactivity of anti-ZIKV MAbs with ZIKV and DENV virus-like particles (VLP) using Luminex assay. ZIKV- and DENV-VLP were conjugated to MagPlex beads (Luminex) and 10,000 beads/mL of these beads mixed with 10 μg/mL to 0.0002 ng/mL anti-ZIKV MAb 102-1, 242-3, 270-12, 289-3, 306-2, 11-3, 278-11, 78-2, and 4G2 for 90 min at room temperature. (B) Western blot analysis of anti-ZIKV MAbs. ZIKV-VLP were heat denatured under nonreduced conditions at 70°C for 5 min. Samples (left, 27 ng; right, 240 ng) were electrophoresed by Wes capillary and detected by 10 μg/mL anti-ZIKV MAb clones. Left, MAb 102-1, 242-3, 270-12, 289-3, 306-2, 78-2 and 11-3. Right, 278-11. M, molecular weight marker. The estimated molecular mass of ZIKV E protein, 55 kDa; prM protein, 23 kDa.

Seven MAbs (102-1, 242-3, 270-12, 289-3, 306-2, 11-3, and 278-11) bound specifically to ZIKV-VLP, and one MAb (78-2) was cross-reactive, binding to both ZIKV- and DENV-VLP (Fig. 1A, Table 1). One MAb (278-11) bound only weakly to ZIKV-VLP. The binding of all MAbs to ZIKV-VLP was at levels greater than a control cross-reactive DENV MAb, 4G2 (Fig. 1A). Five ZIKV-specific MAbs (102-1, 242-3, 270-12, 289-3, and 306-2) demonstrated ZIKV-neutralizing activity (Table 1), with MAb 289-3 displaying the lowest half-maximal inhibitory concentration (IC_50_) values of ZIKV neutralizing antibody titer (7.8 ng/mL).

**TABLE 1:** Summary of characterization of anti-ZIKV MAbs

Clone	Luminex assay		Western reactivity^*b*^	MNT titer, IC_50_, ng/mL^*c*^	Kinetic analysis					
					VLP			E protein		
	Specificity^*a*^	EC_50_, ng/mL			*K_D_*, nM	*k*_*a*_, 1/Ms	*k*_dis_, 1/s	*K_D_*, nM	*k*_*a*_, 1/Ms	*k*_dis_, 1/s
102-1	ZIKV	12.3	E	38.4	0.20	1.30 × 10^5^	2.59 × 10^−5^	1.78	9.67 × 10^4^	1.72 × 10^−4^
242-3	ZIKV	25.1	E	42.8	0.24	2.02 × 10^5^	4.93 × 10^−5^	1.22	6.71 × 10^4^	8.16 × 10^−5^
270-12	ZIKV	46.2	E	37.2	0.31	1.40 × 10^5^	4.27 × 10^−5^	1.04	9.45 × 10^4^	9.80 × 10^−5^
289-3	ZIKV	15.7	ND	7.8	0.30	1.55 × 10^5^	4.58 × 10^−5^	0.29	1.29 × 10^5^	3.68 × 10^−5^
306-2	ZIKV	89.9	E	10,547	0.40	1.60 × 10^5^	6.34 × 10^−5^	0.17	1.17 × 10^5^	2.04 × 10^−5^
78-2	CR	303	ND	ND	0.19	2.21 × 10^5^	4.28 × 10^−5^	0.10	1.92 × 10^5^	1.94 × 10^−5^
278-11	ZIKV	23,800	prM	NT	0.45	1.87 × 10^5^	8.49 × 10^−5^	NT	NT	NT
11-3	ZIKV	6.93	ND	ND	0.57	1.25 × 10^5^	7.13 × 10^−5^	1.53	8.98 × 10^4^	1.37 × 10^−4^

a ZIKV, ZIKV specific; CR, cross-reactive.

b E, Envelope protein; prM, precursor membrane protein. ND, not detected.

c MNT, microneutralization test; NT, not tested.

**FIG 2:**
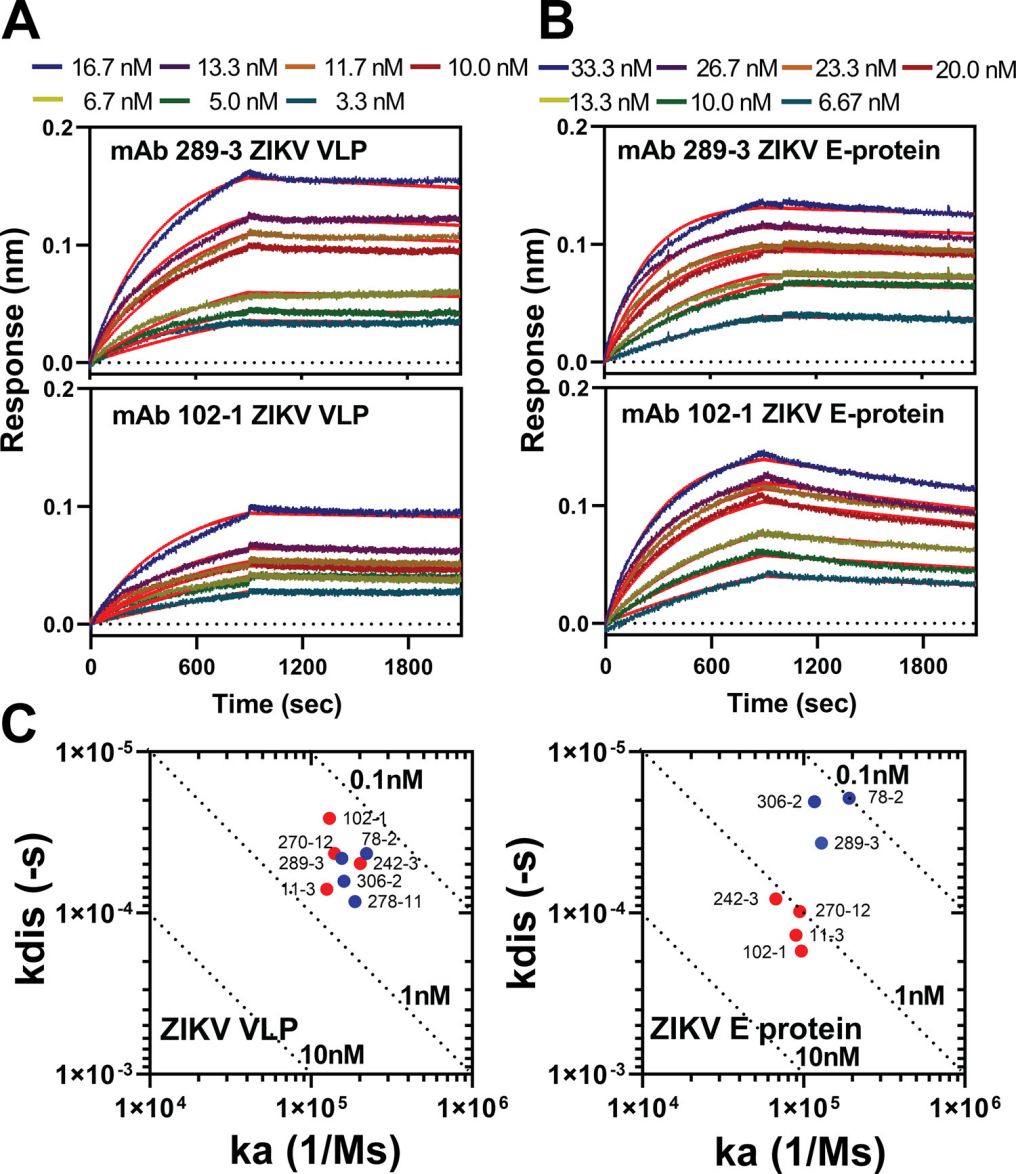
Kinetic analysis of anti-ZIKV MAbs. Kinetic analysis was conducted by Octet HTX (Sartorius). Anti-ZIKV MAbs were conjugated to an amine-reactive 2nd generation (AR2G) biosensor at 0.1 to 0.3 μg/mL, and the association constant (*k_a_*) was measured over 0 to 900 s and dissociation constant (*k*_dis_) for 1,200 s for ZIKV-VLP or E proteins at various concentrations. (A) MAb 289-3 and 102-1 to ZIKV-VLP, 3.3 to 16.7 nM. (B) MAb 289-3 and 102-1 to ZIKV E proteins, 6.67 to 33.3 nM, red line, fitting pattern. (C) *k_a_*/*k*_dis_ plot for anti-ZIKV MAbs to ZIKV-VLP and ZIKV E proteins. Blue, MAb 78-2, 289-3, 306-2 and 278-11; red, MAb 102-1, 242-3. 270-12, and 11-3. *k_a_* and *k*_dis_ values were calculated by two runs, and the average values are shown. Values (nM) for equilibrium dissociation constants (*K_D_*) are shown for each dotted line. *K_D_* = *K_dis_*/*K_a_*.

Four MAbs (102-1, 242-3, 270-12, and 306-2) bound to ZIKV E protein, as determined by Western analysis, while one MAb (278-11) did not bind ZIKV E protein but bound to a 30 kDa protein, identified as ZIKV prM protein (Fig. 1B, Table 1). Three MAbs (289-3, 78-2, and 11-3) did not bind to any ZIKV protein on Western analysis, suggesting that they bind quaternary epitopes. Equilibrium dissociation constants (*K_D_*) ranged from 0.19 to 0.57 nM for MAb binding to ZIKV-VLPs and 0.10 to 1.78 nM for MAb binding to soluble ZIKV E protein. For MAbs 102-1, 242-3, 270-12, and 11-3, *K_D_* for ZIKV-VLPs was lower than *K_D_* for ZIKV E protein (Fig. 2, Table 1).

### Epitope mapping of anti-ZIKV MAbs.

**FIG 3:**
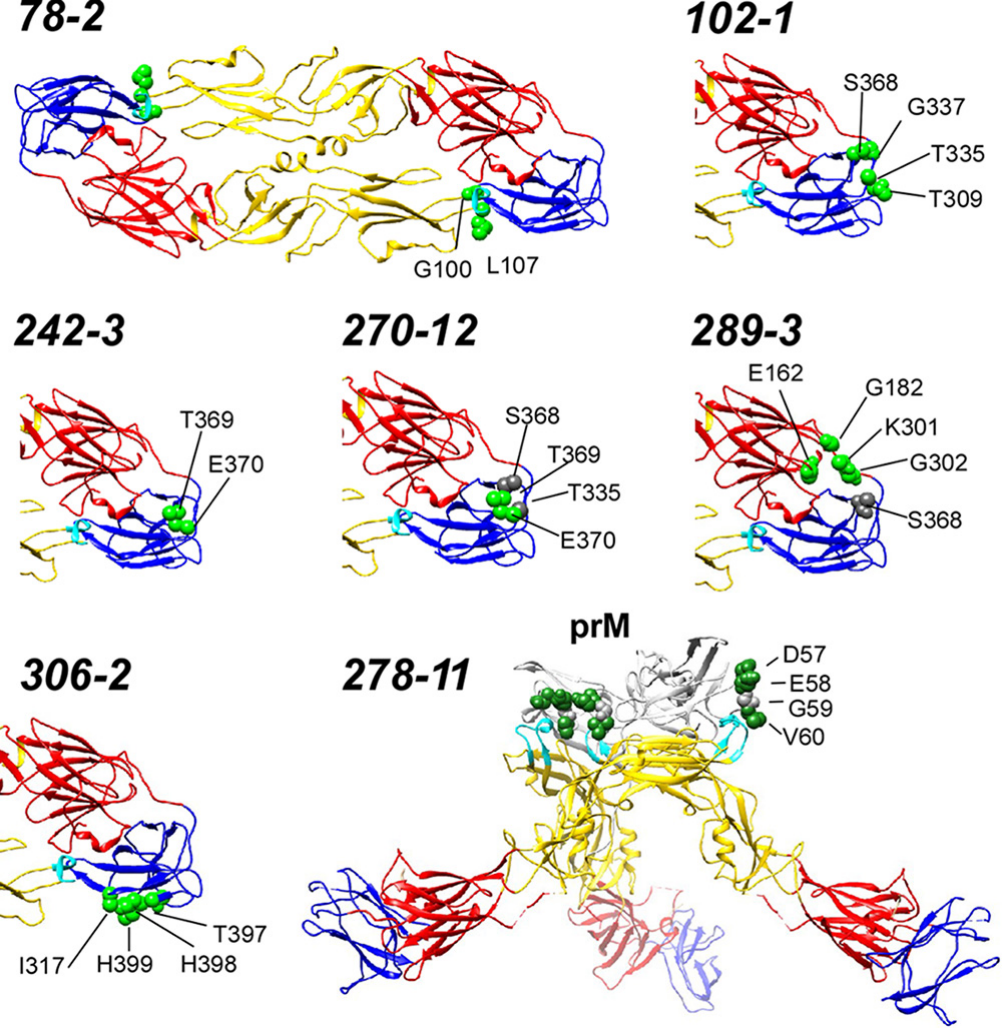
Epitope mapping of anti-ZIKV MAbs. Critical residues (green spheres) for antibody binding are visualized on a crystal structure of the ZIKV E protein dimer (PDB entry 5IRE, 73) or on a cryoelectron microscopy structure of ZIKV precursor membrane prM protein for 278-11 (PDB entry 5U4W, 74). Secondary residues (gray spheres) that may contribute to binding are also shown. Red, E protein domain I; yellow, domain II; blue, domain III. Detailed data are shown in Table 2.

The epitopes recognized by MAbs 102-1, 242-3, 270-12, 289-3, 306-2, 78-2, and 278-11 were mapped by screening for binding against a comprehensive shotgun mutagenesis alanine scanning mutant library covering ZIKV prM/E (Fig. 3, Table 2). Three ZIKV-neutralizing MAbs (102-1, 242-3, and 270-12) bound epitopes in the lateral ridge of domain III. MAb 102-1 recognized an epitope covering residues T309, T335, G337, and S368. The epitope of MAb 270-12 also included residues T335 and S368. MAbs 242-3 and 270-12 also recognized domain III lateral ridge, including residues T369 and E370, in addition to residues T335 and S368. MAb 306-2 bound to an epitope including residues I317, T397, H398, and H399 at the distal end of domain III. Consistent with its cross-reactivity with DENV, MAb 78-2 bound to residues G100 and L107 in the highly conserved fusion loop. The shotgun mutagenesis analysis demonstrated that MAb 289-3 recognized a conformational quaternary epitope spanning domains I and III, including amino acid residues E162, G182, K301, G302, and S368. In addition, MAb 278-11 was confirmed to bind prM protein, with a linear epitope including residues D57, E58, G59, and V60.

**TABLE 2:** Critical amino acid residues on ZIKV E/prM protein important for anti-ZIKV MAb binding^*a*^

Protein	Mutation	Antibody binding reactivity (% WT)						
		278-11^*b*^	78-2	102-1	242-3	270-12	289-3	306-2
prM	D57A	**0.8 (1)**	123.3 (8)	84.5 (1)	91.9 (0)	105.1 (3)	97.92 (0)	122.1 (38)
	E58A	**14.7 (3)**	129.5 (3)	84.0 (9)	96.8 (9)	93.1 (6)	83.9 (18)	117.1 (36)
	G59A	*28.5 (3)*	116.7 (10)	99.4 (3)	94.9 (0)	99.3 (1)	93.6 (2)	127.6 (6)
	V60A	**19.9 (5)**	113.1 (3)	77.6 (4)	97.2 (7)	85.9 (4)	96.7 (16)	100 (36)
E	G100A	95.1 (0)	**18.9 (1)**	72.7 (9)	70.3 (4)	63.6 (2)	72.7 (9)	105.4 (34)
	L107A		**17.3 (0)**	97.1 (19)	121.9 (6)	89.8 (4)	96.8 (27)	98.8 (9)
	E162A	77.8 (21)	101.3 (1)	89.9 (10)	79.6 (11)	84.5 (0)	**4 (0)**	83 (18)
	G182A	85.9 (5)	110.5 (3)	93.2 (8)	85.6 (6)	75.5 (9)	**2.8 (0)**	122.1 (39)
	K301A		82.5 (4)	91.3 (7)	78.7 (14)	79.1 (3)	**7.2 (1)**	64.8 (7)
	G302A		69.2 (14)	89.1 (2)	68.3 (13)	79.1 (0)	**11.5 (2)**	58.8 (10)
	T309A		96.2 (17)	**2.9 (2)**	103.9 (6)	78 (7)	65.2 (8)	72.5 (7)
	I317A		61.2	85.7 (6)	111.8 (40)	106.8 (5)	106.8 (25)	*17.6 (4)*
	T335A		71.6 (13)	*21.5 (2)*	36.2 (15)	*30.1 (1)*	54 (4)	25.5 (25)
	G337A		59.7 (32)	**2.5 (0)**	48.3 (20)	45.1 (1)	40 (11)	127 (0)
	S368A		87.5 (0)	*26.3 (3)*	89.1 (15)	*39.8 (4)*	*23.8 (10)*	71.4 (21)
	T369A		113.0 (2)	66.4 (4)	*34.5 (13)*	*20.8 (7)*	73.6 (7)	79.2 (7)
	E370A		92.7 (6)	84.4 (8)	**12.6 (3)**	**5 (2)**	109.6 (2)	101.6 (5)
	T397A		147	97.3 (6)	110.9 (11)	78.6 (3)	94.7 (20)	**0.9 (2)**
	H398A		92.9 (13)	80.3 (3)	77.2 (9)	76.3 (1)	55 (7)	**3.9 (3)**
	H399A		97.2 (0)	110.1 (1)	127.1 (24)	99.2 (11)	80.2 (10)	*23 (2)*

a MAb binding data for all prM/E clones identified as critical for MAb binding. MAb reactivities for each mutant are expressed as percent binding to wild-type (WT) prM/E, with ranges (half maximum minus minimum values) in parentheses. Values are boldfaced for critical residues and italics for secondary residues. At least two replicate values were obtained for each experiment.

b MAb 278-11 was screened only on a subset of the prM/E library clones that contained the mutations covering the prM protein.

### Antibody allele analysis of anti-ZIKV MAbs.

The rabbit anti-ZIKV MAbs all utilized one of two heavy-chain variable region alleles, IGHV1S40*01 for MAbs 102-1, 289-3, 306-2, 78-2, and 278-11 and IGHV1S45*01 for MAbs 11-3, 242-3, and 270-12 (Table 3). 242-3 and 270-12 recognize the same two amino acids on the domain III lateral ridge (Fig. 3, Table 2). Four alleles for the D region were utilized, IGHD1-1*01, 4-1*01, 7-1*01, and 8-1*01, and two alleles for J region, IGHJ4*01 and IGHJ6*01 (Table 3). The MAbs utilized six alleles of light-chain variable region: five kappa chains, IGKV1S10*01 for MAbs 289-2 and 11-3, IGKV1S32*01 for 278-11, IGKV1S34*01 for 242-3 and 270-12, IGKV1S36*01 for 306-2, and IGKV1S37*01 for 102-1, and one lambda chain, IGLV5S3*01 for 78-2. The J region alleles utilized were IGKJ1-2*01 for kappa and IGLJ5*01 for lambda chain (Table 3).

**TABLE 3:** Summary of anti-ZIKV MAb allele and analysis^*a*^

Clone and chain	V region	SHM (%)		AA mutation (%)		CDR3 AA (*N*)	CDR3 AA sequence	Region	
		DNA	Protein	FWR	CDR^*b*^			D	J
Heavy-chain									
102-1	IGHV1S40*01	9.4	20.6	11.5	57.9	12	IISTGGSHRFNL	IGHD1-1*01	IGHJ4*01
242-3	IGHV1S45*01	5.7	12.1	6.3	35.0	17	ARSSYPDSSGYSYGMDL	IGHD1-1*01	IGHJ6*01
270-12	IGHV1S45*01	5.4	11.1	5.1	35.0	17	ARSSYPDSSGYSYGMDL	IGHD1-1*01	IGHJ6*01
289-3	IGHV1S40*01	10.4	18.6	15.2	33.3	18	ARAIAVGAGYGVGNYFTL	IGHD7-1*01	IGHJ4*01
306-2	IGHV1S40*01	5.8	8.2	3.8	26.3	10	ARHPGTYFTL	IGHD8-1*01	IGHJ4*01
78-2	IGHV1S40*01	8.1	15.3	6.4	50.0	17	ARDLPSFTAPYAGYLRL	IGHD7-1*01	IGHJ4*01
278-11	IGHV1S40*01	10.5	17.9	11.5	47.1	12	ARYNTGGFYYDL	IGHD4-1*01	IGHJ4*01
11-3	IGHV1S45*01	5.4	12.1	10.1	20.0	13	ARGGSTAAAGFNL	IGHD7-1*01	IGHJ4*01
Light-chain									
102-1	IGKV1S37*01	10.7	19.6	11.4	55.6	12	QATDVGGSGRGA		IGKJ1-2*01
242-3	IGKV1S34*01	10.9	22.7	15.2	55.6	12	QTYYDISNYGYA		IGKJ1-2*01
270-12	IGKV1S34*01	11.3	22.7	15.2	55.6	12	QTYYDISNYGYA		IGKJ1-2*01
289-3	IGKV1S10*01	8.2	16.5	11.4	38.9	16	QSYYTSSSNADGSENA		IGKJ1-2*01
306-2	IGKV1S36*01	8.4	16.8	6.3	68.8	12	QTYYYYNKIING		IGKJ1-2*01
78-2	IGLV5S3*01	5.3	8.4	6.3	14.8	13	YTVHATESSLHYV		IGLJ5*01
278-11	IGKV1S32*01	11.2	20.4	17.7	31.6	12	QQGYSSNDADNT		IGKJ1-2*01
11-3	IGKV1S10*01	10.3	19.6	8.9	66.7	13	QCNDYGGTYVPNA		IGKJ1-2*01

a V region, variable region; SHM, somatic hypermutation; CDR, complementarity-determining region; FWR, framework region; D, diversity region; J, joining region; AA, amino acid.

b CDR1 and 2 for heavy-chain and CDR1-3 for light-chain.

### Mutation analysis and CDR3 length of anti-ZIKV MAbs.

**FIG 4:**
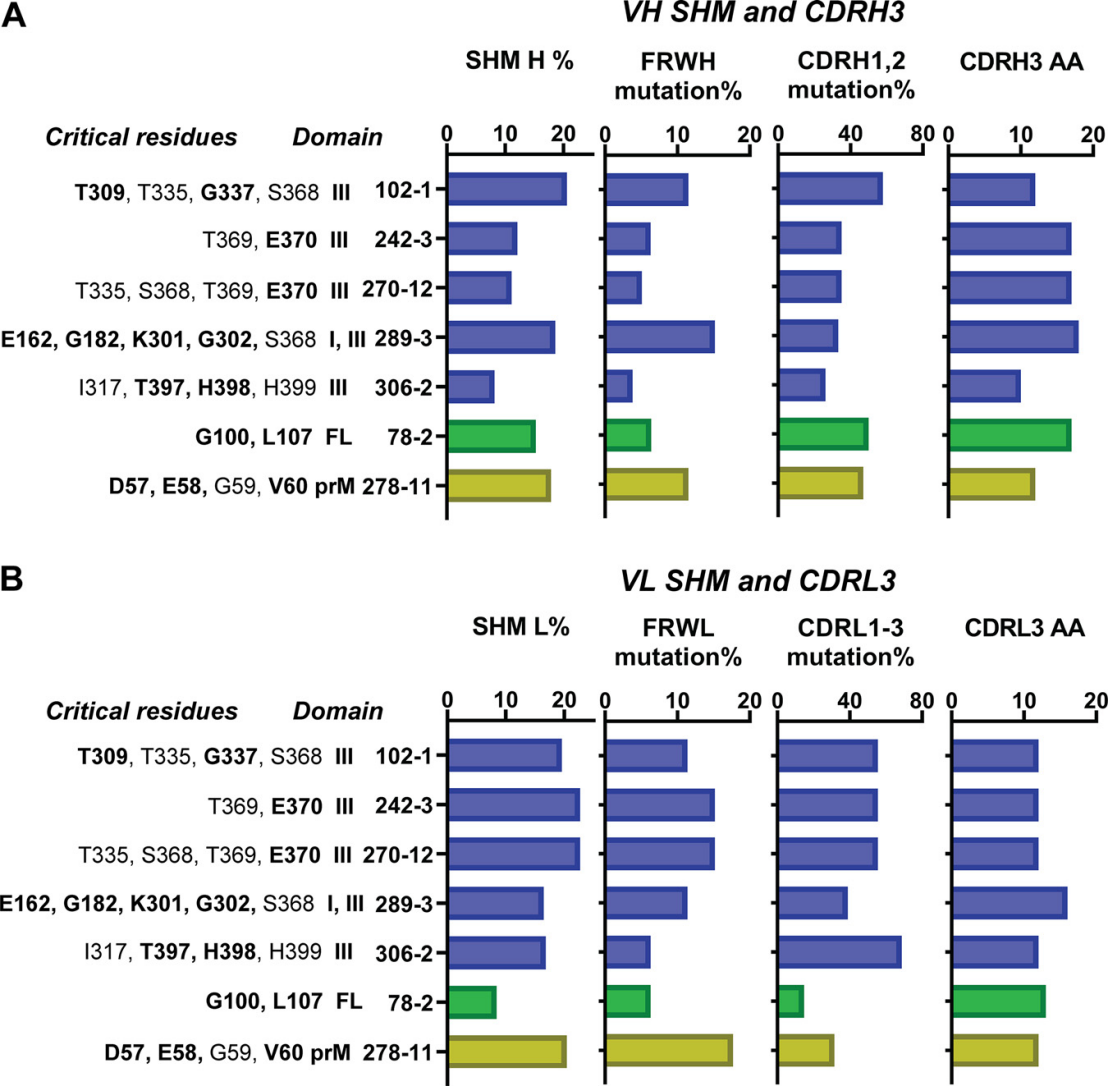
Variable region mutations and CDR3 length of anti-ZIKV MAbs. Somatic hypermutation (SHM), framework region (FWR) mutations, complementarity-determining region (CDR) mutations, and CDR3 amino acid length of anti-ZIKV MAbs. (A) Heavy-chain variable region. (B) Light-chain variable region. Binding ZIKV amino acid residues and ZIKV domains are shown for each MAb (detailed data are in Table 3). Blue bar, MAb epitopes, ZIKV E protein domain III or domain I to III; green bar, MAb epitope, ZIKV E protein fusion loop (FL); yellow bar, MAb epitope, ZIKV precursor membrane (prM) protein. Boldfaced amino acid residues, critical amino acid of ZIKV E protein and prM protein for anti-ZIKV MAb binding (Table 2).

The protein SHM rate for the MAbs varied from 8.2% to 20.6% for heavy-chain and 8.4% to 22.7% for light-chain. The CDR mutation rate ranged from 20.0% to 57.9% for the heavy-chain CDR1 and -2 and 14.8% to 68.8% for the light-chain CDR1 to -3. The FWR was mutated 3.8% to 15.2% for heavy-chain and 6.3% to 17.7% for light-chain (Fig. 4A and B, Table 3). The CDRH3 lengths ranged from 10 to 18 amino acids for heavy-chain and 12 to 16 amino acids for light-chain. We analyzed the neutralizing MAbs to understand the contribution of the heavy-chain mutations and CDRH3 length to specificity and neutralization activity. The two ZIKV-specific neutralizing MAbs that demonstrated the highest SHM/FWR mutation rates were MAb 102-1 (20.6%/11.5%) and MAb 289-3 (18.6%/15.2%), which recognize a quaternary epitope. On the other hand, the neutralizing MAb 306-2, which recognizes an epitope at the distal end of domain III, had the lowest SHM/FWR mutation rate, 8.2%/3.8%. (Fig. 4A and Table 3). Consistent with the high SHM/FWR mutation rates, the quaternary MAb 289-3 had the longest CDR3 length at 18 amino acids. The CDR1,2 mutation rate of MAb 289-3 was 33.3%. The CDR3 length of the MAb 102-1 was 12 amino acid residues, with a high CDRH1,2 mutation rate of 57.9%. The CDR3 length of MAb 306-2 was the shortest among the MAbs at 10 amino acids, with a CDRH1,2 mutation rate of 26.3% (Fig. 4A and Table 3). The other two neutralizing MAbs, 242-3 and 270-12, had similar sequences with an amino acid identity of 99% and similar epitope recognition in the lateral ridge of domain III. SHM/FWR mutation rates (MAb 242-3, 12.1%/6.3%; MAb 270-12, 11.1%/5.1%) and CDR3 lengths (17 amino acids for both) were intermediate between MAbs 289-3 and 306-2. Interestingly, the ZIKV prM-specific non-neutralizing MAb 278-11 also had a high SHM/FWR mutation rate of 17.9%/11.5% and a high CDRH1,2 mutation rate of 47.1%. The CDR3 length of MAb 278-11 was 12 amino acids. In conclusion, among ZIKV-specific neutralizing MAbs, we observed higher somatic mutation and the longest CDR3 length in the MAb recognizing a quaternary epitope. There was no clear trend between epitope mapping information and SHM, FWR mutation, and CDR information for the light-chain variable region, except that the longest CDR3 length (16 amino acids) was also observed in MAb 289-3, which recognizes a quaternary epitope (Fig. 4B and Table 3).

### Correlations among anti-ZIKV MAb variable region mutations, CDR3 length, and antibody binding parameters in ZIKV-neutralizing antibodies.

**FIG 5:**
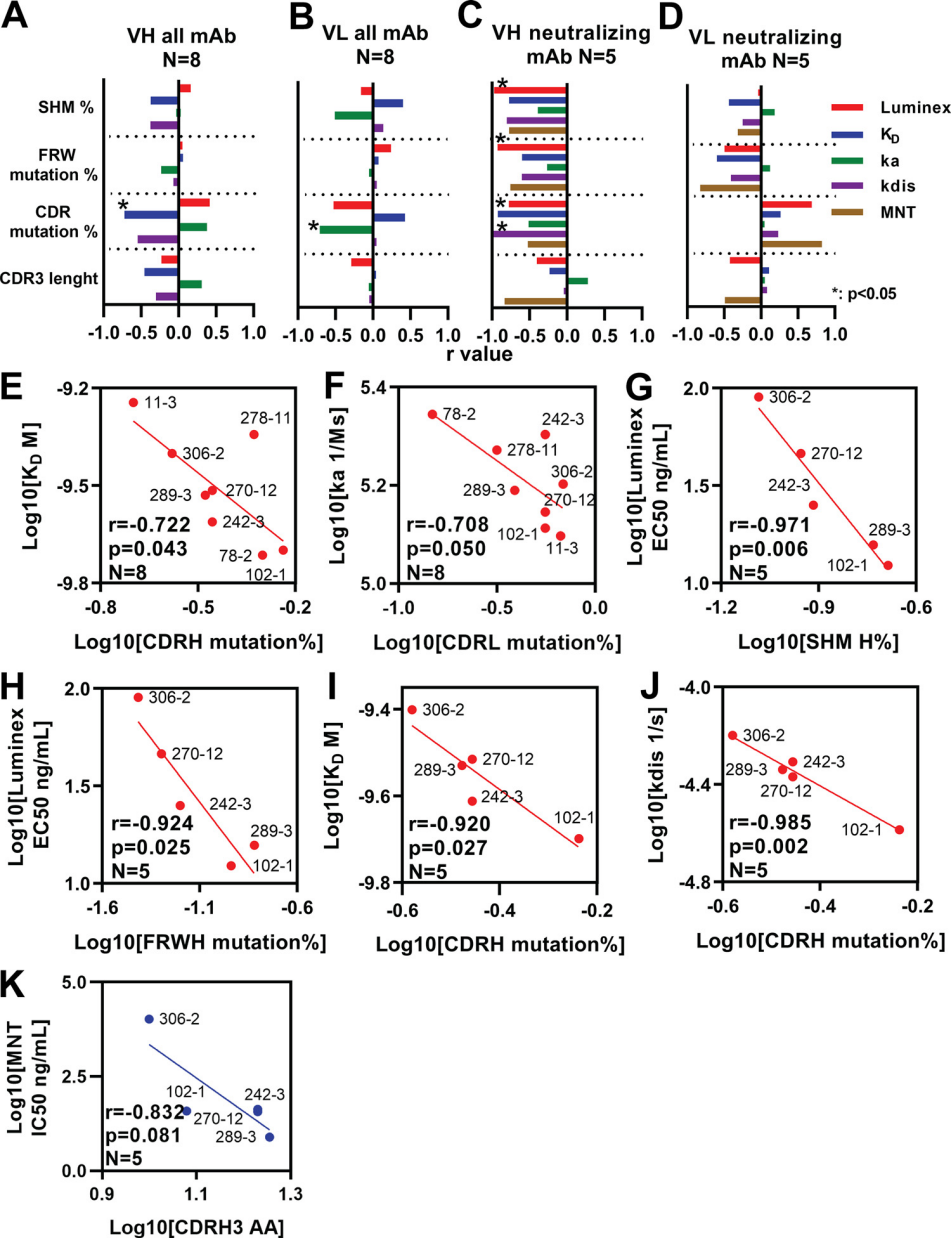
Correlation analysis of anti-ZIKV MAb somatic hypermutations, CDR length, and antibody binding parameters. (A to D) *r* values of correlation analysis between anti-ZIKV MAb somatic hypermutation (SHM), complementarity-determining region (CDR) mutation, framework region (FWR) mutation, CDR3 amino acid length, and antibody binding parameters. (A and B) Binding parameters of all eight anti-ZIKV MAbs, 102-1, 242-3, 270-12, 306-2, 289-3, 78-2, 278-11, and 11-3. (C and D) Anti-ZIKV neutralizing MAbs 102-1, 242-3, 270-12, 306-2, and 289-3. (E to K) Correlation analysis of anti-ZIKV MAb SHM, CDR, FWR mutations, CDR3 length, and binding parameters. (E) Correlation between heavy-chain CDR mutations and equilibrium dissociation constant (*K_D_*) for all anti-ZIKV MAbs. (F) Correlation between light-chain CDR mutations and association constant (*k_a_*) for all anti-ZIKV MAbs. (G) Correlation between heavy-chain SHM rate and Luminex assay EC_50_ value for anti-ZIKV neutralizing MAbs. (H) Correlation between heavy-chain FWR mutation rate and Luminex assay EC_50_ value for anti-ZIKV neutralizing MAbs. (I) Correlation between heavy-chain CDR mutation rate and *K_D_* for anti-ZIKV neutralizing MAbs. (J) Correlation between heavy-chain CDR mutation rate and dissociation constant (*k*_dis_) for anti-ZIKV neutralizing MAbs. (K) Correlation between heavy-chain CDR3 length and microneutralization test (MNT) IC_50_ value for anti-ZIKV neutralizing MAbs. Plot and linear regression curves are shown. Red, *P* < 0.05; blue, *P* > 0.05.

We analyzed the correlation between binding parameters and SHM, CDR, and FWR mutation and CDR3 length for all eight neutralizing and non-neutralizing anti-ZIKV MAbs (Fig. 5A and B and Table 4). Two parameters were negatively correlated: heavy-chain CDR mutation/*K_D_* (*r* = −0.722, *P* = 0.043) (Fig. 5E) and light-chain CDR mutation/association constant (*k_a_*) (*r* = −0.708, *P* = 0.050) (Fig. 5F). There were weak negative to no correlations for other parameters (*r* = −0.546 to −0.427). Focusing on the five ZIKV-neutralizing MAbs, 102-1, 242-3, 270-12, 289-3, and 306-2, we observed negative correlations between binding parameters and heavy-chain SHM, CDRH, and FWR mutation rates (*r* = −0.985 to −0.264) (Fig. 5C, Table 4). There were significant negative correlations between SHM and VLP binding Luminex 50% effective concentration (EC_50_) (*r* = −0.971, *P* = 0.006) (Fig. 5G), FWR mutations and VLP binding Luminex EC_50_ (*r* = −0.924, *P* = 0.025) (Fig. 5H), CDR mutations and *K_D_* (*r* = −0.920, *P* = 0.027) (Fig. 5I), and CDR mutations and dissociation constant (*k*_dis_) (*r* = −0.985, *P* = 0.002) (Fig. 5J). While correlations between CDRH3 length and binding parameters were low, there was a trend for correlation between CDRH3 length and neutralizing antibody EC_50_ values (*r* = −0.831, *P* = 0.081) (Fig. 5K). Overall, correlations were weaker between binding and light-chain parameters, SHM, FWR mutation, and CDR length (*r* = −0.825 to 0.186) (Fig. 5D, Table 4). Contrary to the other observations, CDR mutation parameters showed positive correlations (*r* = 0.049 to 0.826).

**TABLE 4:** Summary of correlation analysis of anti-ZIKV MAb^*a*^

Parameter by chain and MAb	*N*	SHM %		Mutation %				AA length	
				FWR		CDR^*b*^		CDR3	
		*r* value	*P* value	*r* value	*P* value	*r* value	*P* value	*r* value	*P* value
Heavy-chain, all MAbs^*c*^									
Luminex EC_50_ (ng/mL)	8	0.161	0.704	−0.009	0.983	0.414	0.309	-0.229	0.586
*K_D_* (nM)	8	−0.372	0.365	0.059	0.889	−**0.722**^*d*^	0.043	−0.456	0.257
*k_a_* (1/Ms)	8	0.027	0.949	−0.231	0.583	0.379	0.355	0.308	0.458
*k*_dis_ (1/s)	8	−0.375	0.359	−0.068	0.873	−0.546	0.161	−0.303	0.465
Light-chain, all MAbs^*c*^									
Luminex EC_50_ (ng/mL)	8	−0.158	0.709	0.240	0.567	−0.525	0.181	−0.288	0.489
*K_D_* (nM)	8	0.402	0.323	0.075	0.860	0.427	0.291	−0.017	0.968
*k_a_* (1/Ms)	8	−0.507	0.200	−0.051	0.905	**−0.708** ^*d*^	0.050	−0.054	0.900
*k*_dis_ (1/s)	8	0.137	0.747	0.051	0.905	0.049	0.909	−0.047	0.913
Heavy-chain, neutralizing MAb^*d*^									
Luminex EC_50_ (ng/mL)	5	**−0.971** ^*e*^	0.006	**−0.924** ^*e*^	0.025	−0.776	0.123	−0.402	0.502
*K_D_* (nM)	5	−0.772	0.126	−0.597	0.287	**−0.920** ^*e*^	0.027	−0.233	0.706
*k_a_* (1/Ms)	5	−0.388	0.519	−0.264	0.668	−0.511	0.379	0.277	0.652
*k*_dis_ (1/s)	5	−0.803	0.101	−0.603	0.282	**−0.985** ^*e*^	0.002	−0.040	0.950
MNT titer IC_50_ (ng/mL)	5	−0.773	0.126	−0.756	0.139	−0.521	0.368	−0.831	0.081
Light-chain, neutralizing MAb^*d*^									
Luminex EC_50_ (ng/mL)	5	−0.037	0.953	−0.494	0.397	0.689	0.198	−0.421	0.481
*K_D_* (nM)	5	−0.435	0.464	−0.598	0.287	0.265	0.667	0.107	0.864
*k_a_* (1/Ms)	5	0.186	0.764	0.120	0.847	0.049	0.937	−0.009	0.988
*k*_dis_ (1/s)	5	−0.247	0.689	−0.407	0.496	0.231	0.709	0.082	0.896
MNT titer IC_50_ (ng/mL)	5	−0.316	0.605	−0.825	0.085	0.826	0.085	−0.487	0.406

a *K_D_*, equilibrium dissociation constant; *k_a_*, association constant; *k*_dis_, dissociation constant; MNT, micro neutralization test; SHM, somatic hypermutation; FWR, framework region; AA, amino acid; CDR, complementarity-determining region.

b CDR1,2 mutation for heavy-chain and CDR1-3 mutation for light-chain.

c All MAbs included 102-1, 242-3, 270-12, 289-3, 306-2, 78-2, 278-11, and 11-3.

d Neutralizing MAbs included 102-1, 242-3, 270-12, 289-3 and 306-2.

e Bold numbers represent r values that showed *P* value < 0.05, non-blod numbers represent r value that showed *P* value > 0.05.

### Impact of framework amino acids on the binding activity of anti-ZIKV MAbs.

**FIG 6:**
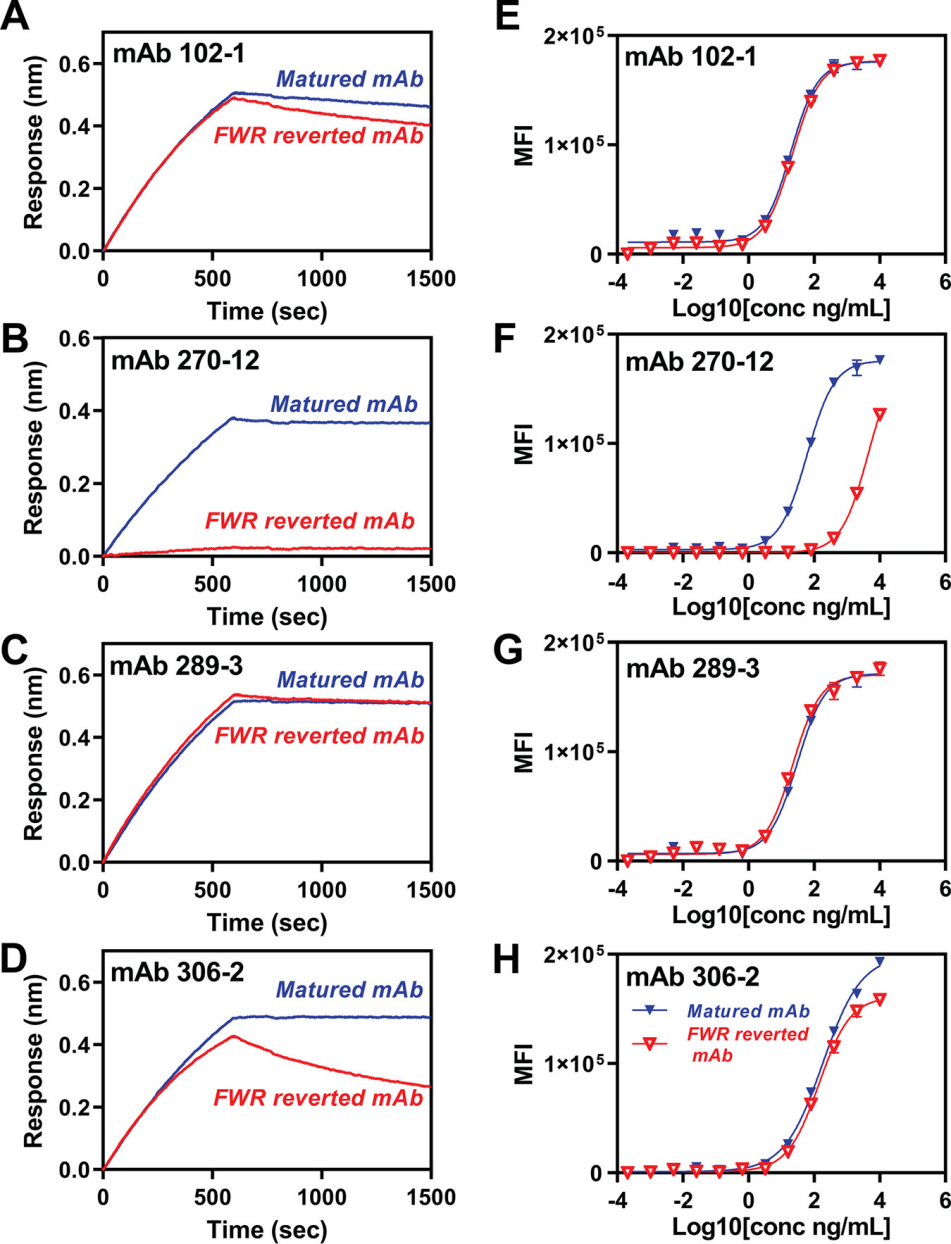
Binding activity of framework region (FWR) amino acid reverted anti-ZIKV MAb. (A to D) Association and dissociation analysis of FWR amino acid reverted anti-ZIKV MAbs by Octet HTX (Sartorius); 2 μg/mL MAbs were captured to protein G biosensor (Sartorius), and 3 μg/mL ZIKV E protein was associated for 600 s and dissociated for 900 s. (E to H) Reactivity of anti-ZIKV MAbs of ZIKV-VLP using Luminex assay. Blue, anti-ZIKV MAbs with matured amino acid; red, anti-ZIKV MAbs with amino acid reverted to allele. (A and E) MAb 102-1. (B and F) MAb 270-12. (C and G) MAb 289-3. (D and H) MAb 306-2.

To understand the impact of the FWR mutations on MAb binding, all FWR amino acids of anti-ZIKV domains I to III and MAb 102-1, 270-12, 289-3, and 306-2 were reverted to the germ line amino acids of the allele and characterized (Fig. 6, Table 5). Reversion of 4 FWR heavy-chain (FWRH) and 12 FWR light-chain (FWRL) amino acids in MAb 270-12 resulted in the loss of binding activity to ZIKV E protein and reduced binding to ZIKV-VLPs (Fig. 6B and F). Reversion of 9/9 and 3/5 amino acids of FWRH/FWRL chains of MAb 102-1 and 306-2, respectively, increased the dissociation rate of ZIKV E protein binding. However, there were no differences in binding to ZIKV-VLPs (Fig. 6A, D, E, and H). Although the highest rate of mutation of FWR H and L chains was observed in MAb 289-3, reversion of 13 FWRH and 9 FWRL amino acids of MAb 289-3 did not alter binding to either ZIKV E protein or ZIKV-VLPs (Fig. 6C and G).

**TABLE 5:** Summary of anti-ZIKV MAb framework amino acid mutations^*a*^

			FWR1 AA		FWR2 AA		FWR3 AA	
Clone and chain	V allele	FWR AA mutation (%)	mutation (*N*)	Mutated AA^*b*^	mutation (*N*)	Mutated AA^*b*^	mutation (*N*)	Mutated AA^*b*^
Heavy-chain								
102-1	IGHV1S40*01	11.5	3	C23>S, T24>K, A25>V	2	M39>I, A54>G	4	S69>N, A71>V, T81>P, Q90>E
270-12	IGHV1S45*01	5.1	1	E2>A	1	I39>M	2	K72>R, Q90>R
289-3	IGHV1S40*01	15.2	3	X2>E, S3>E, T24>K	2	M39>I, L50>P	8	S69>N, G74>R, K80>S, T81>S, T88>A, Q90>E, T92>P, A96>D
306-2	IGHV1S40*01	3.8	0	ND	1	A54>G	2	K80>R, R93>S
Light-chain								
102-1	IGKV1S37*01	11.4	4	S9>A, P10>S, S12>E, N22>K	0	ND	5	E68>D, K77>S, Q86>E, E95>D, T101>S
270-12	IGKV1S34*01	15.2	5	V2>I, S9>A, P10>S, S12>E, N22>K	4	S40>A, P49>R, L52>F, I54>M	3	T69>S, S77>R, E86>Q
289-3	IGKV1S10*01	11.4	5	V2>I, K11>V, D17>G, K22>N, Q24>H	0	ND	4	E68>A, S83>A, S92>T, D93>A
306-2	IGKV1S36*01	6.3	1	P10>S	2	S40>A, K45>R	2	N66>T, R80>G

a AA, amino acid; V allele, variable region allele; FWR, framework region; FWR 1, framework region 1 (framework from N terminal to complementarity-determining region1: CDR1); FWR 2, framework region 2 (framework between CDR1 and CDR2); FWR 3, framework region 3 (framework between CDR2 and CDR3); ND, no mutation detected; X, no amino acid residues from IMGT unique numbering. All amino acids numbers display IMGT unique numbering.

b Mutated amino acids are presented in the following format: allele AA IMGT no.>mutated AA.

## DISCUSSION

We identified and characterized eight unique ZIKV-specific rabbit MAbs with diverse qualities, including epitope specificity, neutralizing activity, and degree of affinity maturation. Rabbits represent an alternative species to generate MAbs with properties similar to those of human MAbs. Rabbits are evolutionarily distinct from mice and other rodents, and rabbit and rodent antibody ontogeny also differ (36). Rabbit antibodies have a long average CDRH3 of 14.8 ± 3.6 amino acids, which is similar to the average human CDRH3 length, 15.3 ± 4.0 amino acids, and longer than the average mouse CDRH3 length of 11.1 ± 2.0 (39). The rabbit CDRH3 length likely contributed to the neutralizing properties of anti-ZIKV MAbs directed to conformational and quaternary epitopes. Human conformational anti-ZIKV-neutralizing antibodies have been described with CDRH3 lengths of 15 to 26 amino acids (39–42). The CDRH3 length of the rabbit anti-ZIKV neutralizing MAbs recognizing conformational epitopes described here had 12 to 18 CDRH3 amino acids. Rabbit immunoglobulin genes also undergo a high degree of variable region rearrangement (39). The SHM rates of the identified rabbit anti-ZIKV MAb genes ranged from 5.4% to 10.5%, compared to SHM rates of published human and mouse anti-ZIKV MAbs of 2.7% to 10.4% (43, 44).

Potently neutralizing ZIKV-specific human MAbs have been described that map to the domain III lateral ridge (43, 45–47), domain II (47, 48), or to complex epitopes spanning multiple domains (49, 50), while fusion loop-specific MAbs are more likely to be cross-reactive with DENV (42). Three of the rabbit MAbs described here map to the domain III lateral ridge, a region that is also targeted by several mouse and human MAbs that have demonstrated ZIKV-neutralizing activity and protective immunity in mouse models, suggesting that this is an immunodominant region for ZIKV-specific neutralizing antibodies in multiple species (45, 47, 48, 51). The epitopes recognized by MAbs 102-1 and 270-12 include residue S368 in the domain III lateral ridge, which has been determined to be an important residue for human ZIKV-specific neutralizing antibodies (52). MAb 242-3 also recognized the domain III lateral ridge, but while S368 was not identified as a critical residue, the adjacent residues T369 and E370 were identified as critical. Other human ZIKV-specific neutralizing antibodies, including ZIKV-116, 7B3, and ZK2B10, recognize domain III lateral ridge epitopes (47, 48, 53) whose residues are overlapping but distinct from those of the rabbit MAbs described here. These results suggest that domain III immunodominant ZIKV-specific epitopes recognized by neutralizing rabbit MAbs are similar to epitopes recognized by human ZIKV-specific MAbs, with the exception of ZIKV-specific neutralizing MAb 306-2, which recognizes a novel conformational epitope at the distal end of domain III.

Potently neutralizing antibodies recognizing complex and quaternary epitopes have been described for a number of viruses, including ZIKV, DENV, and HIV (44, 49, 54, 55). Among the MAbs described here, MAb 289-3 had the strongest neutralizing activity and recognized a quaternary epitope, including critical amino acids in both domains I and III. Previously, a rationally engineered MAb designed to target a quaternary epitope spanning an epitope proximal to the fusion loop was capable of broadly neutralizing ZIKV strains and conferred protection against vertical transmission and fetal mortality in mice (49). Modeling studies suggest that MAbs targeting this region constrain the E protein structure and block fusion (49). Further studies will be required to determine the structure of MAb 289-3 complexed with ZIKV E protein. Three ZIKV-specific human MAbs have recently been described that also span an epitope in domains I and III (44, 50). Alanine-scanning mutagenesis identified the critical residues recognized by two of these MAbs, B11F and A9E, as mapping within domain I alone. Two critical domain I residues of MAb 289-3, E162 and G182, are described as escape mutation sites for MAb A9E (50, 56). We also note that the critical residues K301 (domain III) and G182 (domain I) were also identified by shotgun mutagenesis analysis as critical for binding by the third MAb, protective anti-ZIKV MAb MZ4, which binds a site centered on the E protein domain I/III linker region (44).

CDRH3 length was associated with increased neutralizing antibody activity. A high degree of SHM and relatively long CDRH3 has been associated with the evolution of potent neutralization activity as well as with recognition of complex quaternary epitopes (57). Among the MAbs with ZIKV neutralizing activity, the strength of binding was associated with higher heavy-chain SHM, CDRH, and FWR mutation rates. Our findings were consistent with those for anti-ZIKV EDE1 MAbs C8 and C10, which bind across E protein dimers to strongly neutralize ZIKV (58) and show a high rate of heavy-chain gene SHM, 6.9% and 2.8%, and longer CDRH3 length, 15 and 21 amino acid residues, respectively (42). The association between somatic mutation rate and increased antibody affinity is well established (28, 59, 60). Characterization of MAb 289-3 demonstrates that a high degree of SHM and long CDRH3 can be achieved by ZIKV vaccination and can lead to the evolution of antibodies with potent ZIKV-specific neutralizing activity.

As expected, strong correlations were observed between antibody binding parameters and heavy-chain CDR mutation rate, since CDRs make up the antigen-binding site. Strong correlations were also observed between antibody binding parameters and FWR mutation rate, which was less expected as FWRs likely do not directly bind but provide structural support for the CDRs. FWR mutations may increase antibody flexibility, facilitating CDR contact with epitopes (33, 34). The role of FWR mutations in potency and neutralization of anti-HIV MAbs is variable depending on the specific antibody (61). FWR mutations are important for MAbs against anti-vascular endothelial growth factor, VEGF (34), and FWR mutations have been widely applied, stabilizing the structure of humanized MAbs derived from mice (62). We found that binding activity of MAbs 102-1, 306-2, and 270-12 was impacted by reversion of FWR mutations but that FWR mutations were not essential for binding activity of the most potent MAb, 289-3. This result for MAb 289-3 was different from our expectations, since eight amino acids, the highest number among four MAbs, were changed in heavy-chain FWR3 regions supporting CDR3, and the threonine residue at position 92 (in the international ImMunoGeneTics information system, termed IMGT numbering) (63) was mutated to proline. Although the introduction of a proline residue might be thought likely to perturb the FWR structure, a proline at position 61 was critical for the thermal stability of a broadly neutralizing anti-HIV MAb 3BNC60 (32). Further studies are required to more fully understand the role of FWR amino acids in anti-flavivirus MAb specificity and activity.

In summary, we discovered eight ZIKV-specific MAbs against distinct regions of envelope and prM proteins, including a potent neutralizing MAb that recognized a quaternary epitope spanning domains I and III and a non-neutralizing MAb that recognized a linear epitope on the ZIKV prM protein. Detailed characterization of the rabbit MAbs demonstrated that ZIKV-specific MAbs recognizing conformational and quaternary epitopes on the ZIKV E protein bind with high affinity and are neutralizing. There were significant correlations between the SHM rate, FWR mutation rate, and antibody binding parameters. The higher degree of CDR mutation and SHM, and longest CDRH3, were found in a MAb recognizing a quaternary epitope spanning ZIKV E domains I and III. For some MAbs, reversion of FWR mutations to the germ line allele reduced the affinity of antigen-binding. Thus, we conclude that both SHM and FWR mutations of anti-ZIKV MAbs contribute to antibody affinity, specificity, and functionality.

## MATERIALS AND METHODS

### Ethics.

All procedures were conducted in compliance with the U.S. Department of Agriculture’s Animal Welfare Act (9 CFR Parts 1, 2, and 3), the *Guide for the Care and Use of Laboratory Animals* (64), and the National Institutes of Health, Office of Laboratory Animal Welfare. Whenever possible, procedures in this study were designed to avoid or minimize discomfort, distress, and pain to animals. The animal immunization experiment protocols were approved by the IACUC (International Animal Care and Use Committee) at LabCorp (Denver, PA, USA).

### Antigens and other reagents.

ZIKV (strain; PRVABC59; CDC, Fort Collins, CO) was grown in Vero cells, harvested, purified, and formalin inactivated. These purified inactivated Zika viruses (PIZV) were formulated with aluminum hydroxide. DENV-1-VLP (Nauru/Western Pacific/1974), DENV-2-VLP (Thailand/16681/84), DENV-3-VLP (Sri Lanka D3/H/IMTSSA-SRI/2000/1266), DENV-4-VLP (Dominica/814669/1981), ZIKV-VLP (Suriname Z1106033), and ZIKV E protein (Suriname Z1106033) were purchased from The Native Antigen Company (Oxford, UK). DENV-1 (Nauru/Western Pacific/1974), DENV-2 (Thailand/16681/84), DENV-3 (CH53489), and DENV-4 (TVP/360) inactivated viruses were obtained from Microbix Biosystems (Mississauga, ON, Canada). Anti-flavivirus group antigen-antibody clone D1-4G2-4-15 (4G2) (65) was obtained from Absolute Antibodies (Oxford, UK).

### Rabbit immunization and spleen cell preparation.

Two New Zealand white female rabbits (LabCorp, Denver, PA, USA) were immunized intramuscularly (i.m.) with 5 μg of PIZV plus aluminum hydroxide on days 0, 14, 28, 56, and 95. Both rabbits were boosted i.m. with 5 μg ZIKV-VLP in Freund’s incomplete adjuvant on day 109, followed by intravenous injection of 5 μg ZIKV-VLP on day 130. Splenocytes from rabbits were isolated 4 days after the final boost. The spleen cells were dispersed and subjected to red cell lysis. The cells were frozen in a freezing medium (90% fetal bovine serum and 10% dimethyl sulfoxide) in liquid nitrogen.

### Anti-ZIKV MAb hybridoma generation and clone selection.

Eight hundred million rabbit splenocytes were fused with 400 million fusion partner cells (240E-W2 cells) (66) and plated into 80 96-well plates. The hybridomas were cultured at 37°C, 5% CO_2_. After 14 days, 7,680 multiclonal supernatants were screened by enzyme-linked immunosorbent assay (ELISA) using ZIKV-VLP and ZIKV E protein. A total of 384 clones were positive for ZIKV-VLP alone, and 19 positive multiclones were selected by both ZIKV-VLP and E protein. These multiclones were subcloned by limited dilution, and 155 submonoclones were determined by MAb production; ZIKV neutralizing activity; ELISA against DENV1-4 inactivated virus, ZIKV-VLP, and ZIKV E protein; and *k*_dis_ ranking against ZIKV-VLP using Octet-96 Red (Sartorius, Fremont, CA, USA). We selected 14 clones with high antibody expression for further characterization, nine clones with neutralizing activity and five clones without neutralizing activity.

### DNA sequence analysis of anti-ZIKV MAbs.

Hybridoma cells were collected and lysed for poly(A)^+^ mRNA isolation using poly(A)^+^ RNA isolation kit. Reverse transcription-PCR was conducted using RNA products and synthesized cDNA. First, the rabbit IgG variable region of heavy-chain and full-length light-chain were individually PCR amplified using gene-specific primers. Following gel purification of PCR products, the entire light-chain fragment was cloned into a mammalian light-chain expression vector. Next, the heavy-chain variable fragment was fused with rabbit heavy-chain constant region expression vectors.

### Anti-ZIKV MAb expression and purification.

To express recombinant rabbit monoclonal antibodies, the light- and heavy-chain mammalian expression plasmids were cotransfected into exponentially growing 293-6E cells using lipid-mediated transfection reagent (67). The serum-free culture supernatant was harvested 5 days after transfection by centrifugation. Harvested culture medium was centrifuged to remove cell debris, and the clear supernatant containing secreted monoclonal antibodies was purified through MabSelect SuRe protein A column chromatography (Cytiva, Marlborough, MA, USA). The eluted antibody was dialyzed in phosphate-buffered saline (PBS) buffer, sterile filtered, and adjusted to pH 7.4.

### Antibody expression and purification of anti-ZIKV allele reverted MAbs.

The light and heavy chains of rabbit MAb mammalian expression plasmids were cotransfected into Expi 293 cells systems (Thermo Fisher, Waltham, MA, USA) (68), and the transfected medium was harvested 5 days after transfection with centrifuging. Monoclonal antibodies were purified through protein A Sepharose (Cytiva, Marlborough, MA, USA). The eluted antibody was exchanged to Dulbecco's phosphate-buffered saline, (d-PBS, Gibco, Waltham, MA, USA), using Amicon Ultra (Merck Millipore, Burlington, MA, USA).

### Allele analysis.

Anti-ZIKV MAb allele and CDR3 regions were analyzed by IMGT/V-QUEST (http://www.imgt.org/IMGT_vquest/analysis) and NCBI IGBLAST (https://www.ncbi.nlm.nih.gov/igblast/). SMH rate, FWR mutation, and CDR mutation were calculated by mutated DNA and proteins in the variable region from the allele sequence.

### Neutralization assay.

A 50% tissue culture infective dose (TCID_50_)-based microneutralization test (MNT) was used for the virus-neutralizing activity of MAbs in 96-well plates. ZIKV (PRVABC59; CDC, Fort Collins, CO) grown in Vero cells was used as the challenge virus in the neutralization assay. First, hybridoma supernatants or diluted purified MAbs were incubated with 100 TCID_50_/well of ZIKV for 1.5 h at 37°C 5% CO_2_. Next, the ZIKV-MAb mixture was added to Vero cell monolayers in 96-well plates. The plates were incubated at 37°C 5% CO_2_ for 5 days, and cytopathic effect was scored under light microscopy. Relative infectivity was plotted against MAb concentration, and IC_50_ values were determined as described previously (46).

### Western analysis.

Western blot analysis was conducted by a capillary-based electrophoresis system (69) (Wes; ProteinSimple, Santa Clara, CA, USA). In brief, 27 or 240 ng ZIKV-VLP was denatured at 70°C without reducing agent for 5 min and loaded on a Wes assay plate and electrophoresed. Next, 10 μg/mL anti-ZIKV MAb was charged, followed by Wes horseradish peroxidase-conjugated anti-rabbit secondary antibody. The sample run was analyzed by examining the electropherogram and digital gel image.

### Luminex assay.

The Luminex assay was conducted by FlexMap 3D (Luminex, Austin, TX, USA), and the conjugation of VLP was previously reported (70). Briefly, 65 μg ZIKV and DENV-VLP was conjugated to 1-ethyl-3-[3-dimethylaminopropyl] carbodiimide hydrochloride, ECD/N-hydroxy-sulfo-succinimide, NHS (Thermo Fisher, Waltham, MA, USA), and activated in 12.5 million MagPlex beads (Luminex, Austin, TX, USA) in 50 mM 2-(N-morpholino)ethanesulfonic acid buffer, pH 7.0 or 6.0, for 120 min at room temperature. After conjugation, excess active residues were blocked by sample buffer (1% bovine serum albumin [BSA] in d-PBS) overnight at 4°C. A total of 10,000 ZIKV- and DENV-VLP conjugated beads/mL and anti-ZIKV MAb was incubated at room temperature in sample buffer for 90 min and washed with phosphate-buffered saline plus 0.05% Tween 20 (PBST). After washing, the beads were incubated with 10 μg/mL phycoerythrin-labeled anti-rabbit IgG (Thermo Fisher, Waltham, MA, USA) for 60 min. The beads were washed and mixed with sheath fluid (Luminex, Austin, TX, USA). The plates were measured the fluorescence intensity by FlexMap 3D.

### *K_D_* measurement.

Antibody kinetic analyses were conducted by Octet HTX systems (Sartorius, Fremont, CA, USA). Briefly, 0.1 to 0.3 μg/mL anti-ZIKV MAb was conjugated to an amine-reactive 2nd generation (AR2G) biosensor (Sartorius, Fremont, CA, USA) using EDC/NHS at pH 4.0 or 5.0 in acetic buffer. A volume of 0.1 to 1.0 μg/mL ZIKV-VLP or 0.2 to 2 μg/mL ZIKV E protein in 1× kinetic buffer (Sartorius, Fremont, CA, USA) was associated with anti-ZIKV MAb for 900 s and dissociated for 1,200 s. Kinetic parameters, association constant (*k_a_*), and dissociation constant (*k*_dis_) were analyzed by Octet Data Analysis Software HT (ver. 11.1.2.48 Sartorius, Fremont, CA, USA) with the Langmuir 1:1 binding model. Equilibrium dissociation constants (K_d_) were calculated the following equation: *K_d_* = *k*_dis_/*k*_a_.

### Shotgun mutagenesis epitope mapping.

Epitope mapping by shotgun mutagenesis and alanine-scanning mutagenesis (71) was performed as described previously (47). A ZIKV (ZIKV SPH2015) prM/E alanine scanning mutation library was created, individually changing residues to alanine (or alanine residues to serine). A total of 672 ZIKV prM/E mutants (100% coverage of prM/E) were generated and transfected into HEK-293T cells. Cells were fixed in 4% (vol/vol) paraformaldehyde (Electron Microscopy Sciences, Hatfield, PA, USA) and permeabilized with 0.1% (wt/vol) saponin in d-PBS plus calcium and magnesium (D-PBS++) before incubation with MAbs diluted in D-PBS++, 10% normal goat serum, and 0.1% saponin. Antibodies were detected using 3.75 μg/mL AlexaFluor488-conjugated secondary antibody (Jackson ImmunoResearch Laboratories, West Grove, PA, USA) in 10% normal goat serum with 0.1% saponin. Cells were washed three times with D-PBS++ and 0.1% saponin followed by two washes in d-PBS, and mean cellular fluorescence was detected using a high-throughput iQue flow cytometer (Sartorius, Fremont, CA, USA). MAb reactivities against each mutant prM/E clone were calculated relative to wild-type prM/E reactivity by subtracting the signal from mock-transfected controls and normalizing the wild-type prM/E-transfected controls. The counterscreen strategy facilitates the exclusion of mutants locally misfolded or has an expression defect (72).

### Correlation analysis.

All data were analyzed by GraphPad Prism (Ver.8.0.0, San Diego, CA). Eight anti-ZIKV MAbs (102-1, 242-3, 270-12, 289-3, 306-2, 78-2, 278-11, and 11-3) and five neutralizing anti-ZIKV MAbs bound to E protein domains III and I to III (102-1, 242-3, 270-12, 289-3, and 306-2) were selected for the analysis. We analyzed the correlation between anti-ZIKV MAb variable region mutations (SHM, FWR mutations, and CDR mutations), CDR3 length, and antibody functions (EC_50_ of Luminex assay, *K_D_*, *k_a_*, *k*_dis_ of ZIKV-VLP, and IC_50_ of neutralization). All parameters were converted into log_10_ and compared the correlations.

### Association/dissociation analysis of FWR mutation reverted anti-ZIKV MAb.

Evaluation of anti-ZIKV MAb allele mutation reverted MAb was conducted by Octet HTX (Sartorius, Fremont, CA, USA). Briefly, anti-ZIKV MAbs were diluted to 2 μg/mL in 0.1% BSA-PBST buffer and captured by protein G biosensor (Sartorius, Fremont, CA, USA); 3 μg/mL ZIKV E protein was associated for 600 s and dissociated for 900 s in the same buffer.

## References

[1] Gubler DJ, Markoff L. 2007. Flaviviruses. Lippincott Williams & Wilkins Publishers, Philadelphia, PA.

[2] Foy BD, Kobylinski KC, Chilson Foy JL, Blitvich BJ, Travassos da Rosa A, Haddow AD, Lanciotti RS, Tesh RB. 2011. Probable non-vector-borne transmission of Zika virus, Colorado, USA. Emerg Infect Dis 17:880–882. 10.3201/eid1705.101939. 21529401 PMC3321795

[3] Mlakar J, Korva M, Tul N, Popović M, Poljšak-Prijatelj M, Mraz J, Kolenc M, Resman Rus K, Vesnaver Vipotnik T, Fabjan Vodušek V, Vizjak A, Pižem J, Petrovec M, Avšič Županc T. 2016. Zika virus associated with microcephaly. N Engl J Med 374:951–958. 10.1056/NEJMoa1600651. 26862926

[4] Musso D, Cao-Lormeau VM, Gubler DJ. 2015. Zika virus: following the path of dengue and chikungunya? Lancet 386:243–244. 10.1016/S0140-6736(15)61273-9. 26194519

[5] Duffy MR, Chen TH, Hancock WT, Powers AM, Kool JL, Lanciotti RS, Pretrick M, Marfel M, Holzbauer S, Dubray C, Guillaumot L, Griggs A, Bel M, Lambert AJ, Laven J, Kosoy O, Panella A, Biggerstaff BJ, Fischer M, Hayes EB. 2009. Zika virus outbreak on Yap Island, Federated States of Micronesia. N Engl J Med 360:2536–2543. 10.1056/NEJMoa0805715. 19516034

[6] Cao-Lormeau VM, Musso D. 2014. Emerging arboviruses in the Pacific. Lancet 384:1571–1572. 10.1016/S0140-6736(14)61977-2. 25443481

[7] Musso D, Nilles EJ, Cao-Lormeau VM. 2014. Rapid spread of emerging Zika virus in the Pacific area. Clin Microbiol Infect 20:O595–O596. 10.1111/1469-0691.12707. 24909208

[8] Petersen LR, Jamieson DJ, Powers AM, Honein MA. 2016. Zika virus. N Engl J Med 374:1552–1563. 10.1056/NEJMra1602113. 27028561

[9] Schuler-Faccini L, Ribeiro EM, Feitosa IM, Horovitz DD, Cavalcanti DP, Pessoa A, Doriqui MJ, Neri JI, Neto JM, Wanderley HY, Cernach M, El-Husny AS, Pone MV, Serao CL, Sanseverino MT, Brazilian Medical Genetics Society–Zika Embryopathy Task Force. 2016. Possible association between Zika virus infection and microcephaly–Brazil, 2015. MMWR Morb Mortal Wkly Rep 65:59–62. 10.15585/mmwr.mm6503e2. 26820244

[10] Brasil P, Pereira JP, Jr, Moreira ME, Ribeiro Nogueira RM, Damasceno L, Wakimoto M, Rabello RS, Valderramos SG, Halai UA, Salles TS, Zin AA, Horovitz D, Daltro P, Boechat M, Raja Gabaglia C, Carvalho de Sequeira P, Pilotto JH, Medialdea-Carrera R, Cotrim da Cunha D, Abreu de Carvalho LM, Pone M, Machado Siqueira A, Calvet GA, Rodrigues Baião AE, Neves ES, Nassar de Carvalho PR, Hasue RH, Marschik PB, Einspieler C, Janzen C, Cherry JD, Bispo de Filippis AM, Nielsen-Saines K. 2016. Zika virus infection in pregnant women in Rio de Janeiro. N Engl J Med 375:2321–2334. 10.1056/NEJMoa1602412. 26943629 PMC5323261

[11] Fauci AS, Morens DM. 2016. Zika virus in the Americas–yet another arbovirus threat. N Engl J Med 374:601–604. 10.1056/NEJMp1600297. 26761185

[12] Heymann DL, Hodgson A, Sall AA, Freedman DO, Staples JE, Althabe F, Baruah K, Mahmud G, Kandun N, Vasconcelos PF, Bino S, Menon KU. 2016. Zika virus and microcephaly: why is this situation a PHEIC? Lancet 387:719–721. 10.1016/S0140-6736(16)00320-2. 26876373 PMC7134564

[13] Musso D, Ko AI, Baud D. 2019. Zika virus infection–after the pandemic. N Engl J Med 381:1444–1457. 10.1056/NEJMra1808246. 31597021

[14] Hill SC, Vasconcelos J, Neto Z, Jandondo D, Zé-Zé L, Aguiar RS, Xavier J, Thézé J, Mirandela M, Micolo Cândido AL, Vaz F, Sebastião CDS, Wu CH, Kraemer MUG, Melo A, Schamber-Reis BLF, de Azevedo GS, Tanuri A, Higa LM, Clemente C, da Silva SP, da Silva Candido D, Claro IM, Quibuco D, Domingos C, Pocongo B, Watts AG, Khan K, Alcantara LCJ, Sabino EC, Lackritz E, Pybus OG, Alves MJ, Afonso J, Faria NR. 2019. Emergence of the Asian lineage of Zika virus in Angola: an outbreak investigation. Lancet Infect Dis 19:1138–1147. 10.1016/S1473-3099(19)30293-2. 31559967 PMC6892302

[15] Sapkal GN, Yadav PD, Vegad MM, Viswanathan R, Gupta N, Mourya DT. 2018. First laboratory confirmation on the existence of Zika virus disease in India. J Infect 76:314–317. 10.1016/j.jinf.2017.09.020. 28988896

[16] Phatihattakorn C, Wongsa A, Pongpan K, Anuwuthinawin S, Mungmanthong S, Wongprasert M, Tassaneetrithep B. 2021. Seroprevalence of Zika virus in pregnant women from central Thailand. PLoS One 16:e0257205. 10.1371/journal.pone.0257205. 34516583 PMC8437263

[17] Tun MMN, Mori D, Sabri SB, Kugan O, Shaharom SB, John J, Soe AM, Nwe KM, Dony JF, Inoue S, Morita K, Ahmed K. 2021. Serological evidence of Zika virus infection in febrile patients and healthy blood donors in Sabah, Malaysian Borneo. Am J Trop Med Hyg 106:601–606. 10.4269/ajtmh.21-0802. 34814105 PMC8832921

[18] Ngwe Tun MM, Kyaw AK, Hmone SW, Inoue S, Buerano CC, Soe AM, Moi ML, Hayasaka D, Thu HM, Hasebe F, Thant KZ, Morita K. 2018. Detection of Zika virus infection in Myanmar. Am J Trop Med Hyg 98:868–871. 10.4269/ajtmh.17-0708. 29363460 PMC5930904

[19] Gobillot TA, Kikawa C, Lehman DA, Kinuthia J, Drake AL, Jaoko W, Mandaliya K, John-Stewart G, McClelland RS, Overbaugh J. 2020. Zika virus circulates at low levels in western and coastal Kenya. J Infect Dis 222:847–852. 10.1093/infdis/jiaa158. 32242626 PMC7399697

[20] Diarra I, Nurtop E, Sangaré AK, Sagara I, Pastorino B, Sacko S, Zeguimé A, Coulibaly D, Fofana B, Gallian P, Priet S, Drexler JF, Failloux AB, Dabo A, Thera MA, Djimdé A, Kouriba B, Cauchemez S, de Lamballerie X, Hozé N, Doumbo OK. 2020. Zika virus circulation in Mali. Emerg Infect Dis 26:945–952. 10.3201/eid2605.191383. 32310065 PMC7181926

[21] Mengesha Tsegaye M, Beyene B, Ayele W, Abebe A, Tareke I, Sall A, Yactayo S, Shibeshi ME, Staples E, Belay D, Lilay A, Alemu A, Alemu E, Kume A, H/Mariam A, Ronveaux O, Tefera M, Kassa W, Bekele Weyessa A, Jima D, Kebede A, Tayachew A. 2018. Sero-prevalence of yellow fever and related Flavi viruses in Ethiopia: a public health perspective. BMC Public Health 18:1011. 10.1186/s12889-018-5726-9. 30107830 PMC6092792

[22] Lazear HM, Diamond MS. 2016. Zika virus: new clinical syndromes and its emergence in the Western Hemisphere. J Virol 90:4864–4875. 10.1128/JVI.00252-16. 26962217 PMC4859708

[23] Dai L, Song J, Lu X, Deng YQ, Musyoki AM, Cheng H, Zhang Y, Yuan Y, Song H, Haywood J, Xiao H, Yan J, Shi Y, Qin CF, Qi J, Gao GF. 2016. Structures of the Zika virus envelope protein and its complex with a flavivirus broadly protective antibody. Cell Host Microbe 19:696–704. 10.1016/j.chom.2016.04.013. 27158114

[24] Pierson TC, Diamond MS. 2012. Degrees of maturity: the complex structure and biology of flaviviruses. Curr Opin Virol 2:168–175. 10.1016/j.coviro.2012.02.011. 22445964 PMC3715965

[25] Roth DB. 2014. V(D)J recombination: mechanism, errors, and fidelity. Microbiol Spectr 2. 10.1128/microbiolspec.MDNA3-0041-2014. PMC5089068 26104458

[26] Mishra AK, Mariuzza RA. 2018. Insights into the structural basis of antibody affinity maturation from next-generation sequencing. Front Immunol 9:117. 10.3389/fimmu.2018.00117. 29449843 PMC5799246

[27] Feng Y, Seija N, Di Noia JM, Martin A. 2020. AID in antibody diversification: there and back again. Trends Immunol 41:586–600. 10.1016/j.it.2020.04.009. 32434680 PMC7183997

[28] Xu JL, Davis MM. 2000. Diversity in the CDR3 region of V(H) is sufficient for most antibody specificities. Immunity 13:37–45. 10.1016/S1074-7613(00)00006-6. 10933393

[29] Morea V, Tramontano A, Rustici M, Chothia C, Lesk AM. 1998. Conformations of the third hypervariable region in the VH domain of immunoglobulins. J Mol Biol 275:269–294. [9466909]. 10.1006/jmbi.1997.1442. 9466909

[30] Chothia C, Novotný J, Bruccoleri R, Karplus M. 1985. Domain association in immunoglobulin molecules. The packing of variable domains. J Mol Biol 186:651–663. [4093982]. 10.1016/0022-2836(85)90137-8. 4093982

[31] Herold EM, John C, Weber B, Kremser S, Eras J, Berner C, Deubler S, Zacharias M, Buchner J. 2017. Determinants of the assembly and function of antibody variable domains. Sci Rep 7:12276. 10.1038/s41598-017-12519-9. 28947772 PMC5613017

[32] Klein F, Diskin R, Scheid JF, Gaebler C, Mouquet H, Georgiev IS, Pancera M, Zhou T, Incesu RB, Fu BZ, Gnanapragasam PN, Oliveira TY, Seaman MS, Kwong PD, Bjorkman PJ, Nussenzweig MC. 2013. Somatic mutations of the immunoglobulin framework are generally required for broad and potent HIV-1 neutralization. Cell 153:126–138. 10.1016/j.cell.2013.03.018. 23540694 PMC3792590

[33] Henderson R, Watts BE, Ergin HN, Anasti K, Parks R, Xia SM, Trama A, Liao HX, Saunders KO, Bonsignori M, Wiehe K, Haynes BF, Alam SM. 2019. Selection of immunoglobulin elbow region mutations impacts interdomain conformational flexibility in HIV-1 broadly neutralizing antibodies. Nat Commun 10:654. 10.1038/s41467-019-08415-7. 30737386 PMC6368608

[34] Koenig P, Lee CV, Walters BT, Janakiraman V, Stinson J, Patapoff TW, Fuh G. 2017. Mutational landscape of antibody variable domains reveals a switch modulating the interdomain conformational dynamics and antigen binding. Proc Natl Acad Sci USA 114:E486–E495. 10.1073/pnas.1613231114. 28057863 PMC5278476

[35] Zhang YF, Ho M. 2017. Humanization of rabbit monoclonal antibodies via grafting combined Kabat/IMGT/Paratome complementarity-determining regions: rationale and examples. MAbs 9:419–429. 10.1080/19420862.2017.1289302. 28165915 PMC5384799

[36] Weber J, Peng H, Rader C. 2017. From rabbit antibody repertoires to rabbit monoclonal antibodies. Exp Mol Med 49:e305. 10.1038/emm.2017.23. 28336958 PMC5382564

[37] Hoang LL, Tang P, Hicks DG, Chen H, Yang Q, Haas TS, Bremer RE, Tacha D. 2014. A new rabbit monoclonal E-cadherin antibody [EP700Y] shows higher sensitivity than mouse monoclonal E-cadherin [HECD-1] antibody in breast ductal carcinomas and does not stain breast lobular carcinomas. Appl Immunohistochem Mol Morphol 22:606–612. 10.1097/PAI.0b013e3182a4edef. 24569788

[38] Pope ME, Soste MV, Eyford BA, Anderson NL, Pearson TW. 2009. Anti-peptide antibody screening: selection of high affinity monoclonal reagents by a refined surface plasmon resonance technique. J Immunol Methods 341:86–96. 10.1016/j.jim.2008.11.004. 19041872

[39] Lavinder JJ, Hoi KH, Reddy ST, Wine Y, Georgiou G. 2014. Systematic characterization and comparative analysis of the rabbit immunoglobulin repertoire. PLoS One 9:e101322. 10.1371/journal.pone.0101322. 24978027 PMC4076286

[40] Barba-Spaeth G, Dejnirattisai W, Rouvinski A, Vaney MC, Medits I, Sharma A, Simon-Lorière E, Sakuntabhai A, Cao-Lormeau VM, Haouz A, England P, Stiasny K, Mongkolsapaya J, Heinz FX, Screaton GR, Rey FA. 2016. Structural basis of potent Zika-dengue virus antibody cross-neutralization. Nature 536:48–53. 10.1038/nature18938. 27338953

[41] Dejnirattisai W, Wongwiwat W, Supasa S, Zhang X, Dai X, Rouvinski A, Jumnainsong A, Edwards C, Quyen NTH, Duangchinda T, Grimes JM, Tsai WY, Lai CY, Wang WK, Malasit P, Farrar J, Simmons CP, Zhou ZH, Rey FA, Mongkolsapaya J, Screaton GR. 2015. A new class of highly potent, broadly neutralizing antibodies isolated from viremic patients infected with dengue virus. Nat Immunol 16:170–177. 10.1038/ni.3058. 25501631 PMC4445969

[42] Dussupt V, Modjarrad K, Krebs SJ. 2020. Landscape of monoclonal antibodies targeting Zika and Dengue: therapeutic solutions and critical insights for vaccine development. Front Immunol 11:621043. 33664734 10.3389/fimmu.2020.621043 PMC7921836

[43] Wang J, Bardelli M, Espinosa DA, Pedotti M, Ng TS, Bianchi S, Simonelli L, Lim EXY, Foglierini M, Zatta F, Jaconi S, Beltramello M, Cameroni E, Fibriansah G, Shi J, Barca T, Pagani I, Rubio A, Broccoli V, Vicenzi E, Graham V, Pullan S, Dowall S, Hewson R, Jurt S, Zerbe O, Stettler K, Lanzavecchia A, Sallusto F, Cavalli A, Harris E, Lok SM, Varani L, Corti D. 2017. A human bi-specific antibody against Zika virus with high therapeutic potential. Cell 171:229–241. 10.1016/j.cell.2017.09.002. 28938115 PMC5673489

[44] Dussupt V, Sankhala RS, Gromowski GD, Donofrio G, De La Barrera RA, Larocca RA, Zaky W, Mendez-Rivera L, Choe M, Davidson E, McCracken MK, Brien JD, Abbink P, Bai H, Bryan AL, Bias CH, Berry IM, Botero N, Cook T, Doria-Rose NA, Escuer AGI, Frimpong JA, Geretz A, Hernandez M, Hollidge BS, Jian N, Kabra K, Leggat DJ, Liu J, Pinto AK, Rutvisuttinunt W, Setliff I, Tran U, Townsley S, Doranz BJ, Rolland M, McDermott AB, Georgiev IS, Thomas R, Robb ML, Eckels KH, Barranco E, Koren M, Smith DR, Jarman RG, George SL, Stephenson KE, Barouch DH, Modjarrad K, Michael NL, et al. 2020. Potent Zika and dengue cross-neutralizing antibodies induced by Zika vaccination in a dengue-experienced donor. Nat Med 26:228–235. 10.1038/s41591-019-0746-2. 32015557 PMC7018608

[45] Zhao H, Fernandez E, Dowd KA, Speer SD, Platt DJ, Gorman MJ, Govero J, Nelson CA, Pierson TC, Diamond MS, Fremont DH. 2016. Structural basis of Zika virus-specific antibody protection. Cell 166:1016–1027. 10.1016/j.cell.2016.07.020. 27475895 PMC4983199

[46] Stettler K, Beltramello M, Espinosa DA, Graham V, Cassotta A, Bianchi S, Vanzetta F, Minola A, Jaconi S, Mele F, Foglierini M, Pedotti M, Simonelli L, Dowall S, Atkinson B, Percivalle E, Simmons CP, Varani L, Blum J, Baldanti F, Cameroni E, Hewson R, Harris E, Lanzavecchia A, Sallusto F, Corti D. 2016. Specificity, cross-reactivity, and function of antibodies elicited by Zika virus infection. Science 353:823–826. 10.1126/science.aaf8505. 27417494

[47] Sapparapu G, Fernandez E, Kose N, Bin C, Fox JM, Bombardi RG, Zhao H, Nelson CA, Bryan AL, Barnes T, Davidson E, Mysorekar IU, Fremont DH, Doranz BJ, Diamond MS, Crowe JE. 2016. Neutralizing human antibodies prevent Zika virus replication and fetal disease in mice. Nature 540:443–447. 10.1038/nature20564. 27819683 PMC5583716

[48] Niu X, Zhao L, Qu L, Yao Z, Zhang F, Yan Q, Zhang S, Liang R, Chen P, Luo J, Xu W, Lv H, Liu X, Lei H, Yi C, Li P, Wang Q, Wang Y, Yu L, Zhang X, Bryan LA, Davidson E, Doranz JB, Feng L, Pan W, Zhang F, Chen L. 2019. Convalescent patient-derived monoclonal antibodies targeting different epitopes of E protein confer protection against Zika virus in a neonatal mouse model. Emerg Microbes Infect 8:749–759. 10.1080/22221751.2019.1614885. 31130109 PMC6542155

[49] Tharakaraman K, Watanabe S, Chan KR, Huan J, Subramanian V, Chionh YH, Raguram A, Quinlan D, McBee M, Ong EZ, Gan ES, Tan HC, Tyagi A, Bhushan S, Lescar J, Vasudevan SG, Ooi EE, Sasisekharan R. 2018. Rational engineering and characterization of an mAb that neutralizes Zika virus by targeting a mutationally constrained quaternary epitope. Cell Host Microbe 23:618–627. 10.1016/j.chom.2018.04.004. 29746833 PMC6018055

[50] Graham SD, Tu HA, McElvany BD, Graham NR, Grinyo A, Davidson E, Doranz BJ, Diehl SA, de Silva AM, Markmann AJ. 2021. A novel antigenic site spanning domains I and III of the Zika virus envelope glycoprotein is the target of strongly neutralizing human monoclonal antibodies. J Virol 95:e02423-20. 10.1128/JVI.02423-20. 33597214 PMC8104094

[51] Wang L, Wang R, Wang L, Ben H, Yu L, Gao F, Shi X, Yin C, Zhang F, Xiang Y, Zhang L. 2019. Structural basis for neutralization and protection by a Zika virus-specific human antibody. Cell Rep 26:3360–3368. 10.1016/j.celrep.2019.02.062. 30893607

[52] Bailey MJ, Broecker F, Freyn AW, Choi A, Brown JA, Fedorova N, Simon V, Lim JK, Evans MJ, García-Sastre A, Palese P, Tan GS. 2019. Human monoclonal antibodies potently neutralize Zika virus and select for escape mutations on the lateral ridge of the envelope protein. J Virol 93:e00405-19. 10.1128/JVI.00405-19. 31043537 PMC6600209

[53] Gao F, Lin X, He L, Wang R, Wang H, Shi X, Zhang F, Yin C, Zhang L, Zhu J, Yu L. 2019. Development of a potent and protective germline-like antibody lineage against Zika virus in a convalescent human. Front Immunol 10:2424. 10.3389/fimmu.2019.02424. 31708914 PMC6821881

[54] Wahala WM, Silva AM. 2011. The human antibody response to dengue virus infection. Viruses 3:2374–2395. 10.3390/v3122374. 22355444 PMC3280510

[55] Burton DR, Hangartner L. 2016. Broadly neutralizing antibodies to HIV and their role in vaccine design. Annu Rev Immunol 34:635–659. 10.1146/annurev-immunol-041015-055515. 27168247 PMC6034635

[56] Collins MH, Tu HA, Gimblet-Ochieng C, Liou GA, Jadi RS, Metz SW, Thomas A, McElvany BD, Davidson E, Doranz BJ, Reyes Y, Bowman NM, Becker-Dreps S, Bucardo F, Lazear HM, Diehl SA, de Silva AM. 2019. Human antibody response to Zika targets type-specific quaternary structure epitopes. JCI Insight 4:e124588. 10.1172/jci.insight.124588. PMC6538335 30996133

[57] Mascola JR, Haynes BF. 2013. HIV-1 neutralizing antibodies: understanding nature's pathways. Immunol Rev 254:225–244. 10.1111/imr.12075. 23772623 PMC3738265

[58] Swanstrom JA, Plante JA, Plante KS, Young EF, McGowan E, Gallichotte EN, Widman DG, Heise MT, de Silva AM, Baric RS. 2016. Dengue virus envelope dimer epitope monoclonal antibodies isolated from dengue patients are protective against Zika virus. mBio 7:e01123-16. 10.1128/mBio.01123-16. 27435464 PMC4958264

[59] Sok D, Laserson U, Laserson J, Liu Y, Vigneault F, Julien J-P, Briney B, Ramos A, Saye KF, Le K, Mahan A, Wang S, Kardar M, Yaari G, Walker LM, Simen BB, St John EP, Chan-Hui P-Y, Swiderek K, Kleinstein SH, Kleinstein SH, Alter G, Seaman MS, Chakraborty AK, Koller D, Wilson IA, Church GM, Burton DR, Poignard P. 2013. The effects of somatic hypermutation on neutralization and binding in the PGT121 family of broadly neutralizing HIV antibodies. PLoS Pathog 9:e1003754. 10.1371/journal.ppat.1003754. 24278016 PMC3836729

[60] Doria-Rose NA, Joyce MG. 2015. Strategies to guide the antibody affinity maturation process. Curr Opin Virol 11:137–147. 10.1016/j.coviro.2015.04.002. 25913818 PMC4456294

[61] Georgiev IS, Rudicell RS, Saunders KO, Shi W, Kirys T, McKee K, O'Dell S, Chuang GY, Yang ZY, Ofek G, Connors M, Mascola JR, Nabel GJ, Kwong PD. 2014. Antibodies VRC01 and 10E8 neutralize HIV-1 with high breadth and potency even with Ig-framework regions substantially reverted to germline. J Immunol 192:1100–1106. 10.4049/jimmunol.1302515. 24391217 PMC4140862

[62] Safdari Y, Farajnia S, Asgharzadeh M, Khalili M. 2013. Antibody humanization methods–a review and update. Biotechnol Genet Eng Rev 29:175–186. 10.1080/02648725.2013.801235. 24568279

[63] Lefranc MP, Pommié C, Ruiz M, Giudicelli V, Foulquier E, Truong L, Thouvenin-Contet V, Lefranc G. 2003. IMGT unique numbering for immunoglobulin and T cell receptor variable domains and Ig superfamily V-like domains. Dev Comp Immunol 27:55–77. 10.1016/S0145-305X(02)00039-3. 12477501

[64] National Research Council. 2011. Guide for the care and use of laboratory animals, 8th ed. National Academies Press, Washington, DC.

[65] Halstead SB, Venkateshan CN, Gentry MK, Larsen LK. 1984. Heterogeneity of infection enhancement of dengue 2 strains by monoclonal antibodies. J Immunol 132:1529–1532. [6607288]. 6607288

[66] Huang Y, Gu B, Wu R, Zhang J, Li Y, Zhang M. 2007. Development of a rabbit monoclonal antibody group against Smads and immunocytochemical study of human and mouse embryonic stem cells. Hybridoma (Larchmt) 26:387–391. 10.1089/hyb.2007.0517. 18158783

[67] Jäger V, Groenewold J, Krüger D, Schwarz D, Vollmer V. 2015. High-titer expression of recombinant antibodies by transiently transfected HEK 293-6E cell cultures. BMC Proc 9:P40. 10.1186/1753-6561-9-S9-P40.

[68] Taki S, Kamada H, Inoue M, Nagano K, Mukai Y, Higashisaka K, Yoshioka Y, Tsutsumi Y, Tsunoda S. 2015. A novel bispecific antibody against human CD3 and ephrin receptor A10 for breast cancer therapy. PLoS One 10:e0144712. 10.1371/journal.pone.0144712. 26678395 PMC4682974

[69] Harris VM. 2015. Protein detection by simple Western analysis. Methods Mol Biol 1312:465–468. 10.1007/978-1-4939-2694-7_47. 26044028

[70] Nascimento EJM, Norwood B, Parker A, Braun R, Kpamegan E, Dean HJ. 2021. Development and characterization of a multiplex assay to quantify complement-fixing antibodies against Dengue virus. Int J Mol Sci 22:12004. 10.3390/ijms222112004. 34769432 PMC8584793

[71] Davidson E, Doranz BJ. 2014. A high-throughput shotgun mutagenesis approach to mapping B-cell antibody epitopes. Immunology 143:13–20. 10.1111/imm.12323. 24854488 PMC4137951

[72] Paes C, Ingalls J, Kampani K, Sulli C, Kakkar E, Murray M, Kotelnikov V, Greene TA, Rucker JB, Doranz BJ. 2009. Atomic-level mapping of antibody epitopes on a GPCR. J Am Chem Soc 131:6952–6954. 10.1021/ja900186n. 19453194 PMC2943208

[73] Sirohi D, Chen Z, Sun L, Klose T, Pierson TC, Rossmann MG, Kuhn RJ. 2016. The 3.8 Å resolution cryo-EM structure of Zika virus. Science 352:467–470. 10.1126/science.aaf5316. 27033547 PMC4845755

[74] Prasad VM, Miller AS, Klose T, Sirohi D, Buda G, Jiang W, Kuhn RJ, Rossmann MG. 2017. Structure of the immature Zika virus at 9 Å resolution. Nature Structural & Molecular Biology 24:184–186. 10.1038/nsmb.3352. PMC5296287 28067914

